# Systematic Analysis of Gene Expression Differences between Left and Right Atria in Different Mouse Strains and in Human Atrial Tissue

**DOI:** 10.1371/journal.pone.0026389

**Published:** 2011-10-19

**Authors:** Peter C. Kahr, Ilaria Piccini, Larissa Fabritz, Boris Greber, Hans Schöler, Hans H. Scheld, Andreas Hoffmeier, Nigel A. Brown, Paulus Kirchhof

**Affiliations:** 1 Department of Cardiology and Angiology, University Hospital Münster, Münster, Germany; 2 Division of Biomedical Sciences, St. George's University of London, London, United Kingdom; 3 Department of Cell and Developmental Biology, Max Planck Institute for Molecular Biomedicine, Münster, Germany; 4 Department of Thoracic and Cardiovascular Surgery, University Hospital Münster, Münster, Germany; Cardiovascular Research Institute Maastricht, Maastricht University, The Netherlands

## Abstract

**Background:**

Normal development of the atria requires left-right differentiation during embryonic development. Reduced expression of *Pitx2c* (paired-like homeodomain transcription factor 2, isoform c), a key regulator of left-right asymmetry, has recently been linked to atrial fibrillation. We therefore systematically studied the molecular composition of left and right atrial tissue in adult murine and human atria.

**Methods:**

We compared left and right atrial gene expression in healthy, adult mice of different strains and ages by employing whole genome array analyses on freshly frozen atrial tissue. Selected genes with enriched expression in either atrium were validated by RT-qPCR and Western blot in further animals and in shock-frozen left and right atrial appendages of patients undergoing open heart surgery.

**Results:**

We identified 77 genes with preferential expression in one atrium that were common in all strains and age groups analysed. Independent of strain and age, *Pitx2c* was the gene with the highest enrichment in left atrium, while *Bmp10*, a member of the TGFβ family, showed highest enrichment in right atrium. These differences were validated by RT-qPCR in murine and human tissue. Western blot showed a 2-fold left-right concentration gradient in PITX2 protein in adult human atria. Several of the genes and gene groups enriched in left atria have a known biological role for maintenance of healthy physiology, specifically the prevention of atrial pathologies involved in atrial fibrillation, including membrane electrophysiology, metabolic cellular function, and regulation of inflammatory processes. Comparison of the array datasets with published array analyses in heterozygous *Pitx2c^+/−^* atria suggested that approximately half of the genes with left-sided enrichment are regulated by *Pitx2c*.

**Conclusions:**

Our study reveals systematic differences between left and right atrial gene expression and supports the hypothesis that *Pitx2c* has a functional role in maintaining “leftness” in the atrium in adult murine and human hearts.

## Introduction

Atrial fibrillation is the most common sustained arrhythmia [1], [2], [3], [4]. Several forms of atrial damage, including electrical and structural “remodelling”, are the main cause of atrial fibrillation [5], [6]. It is generally accepted that atrial fibrillation is mainly a left atrial disease. This old concept has been reinforced by the development of ablation techniques to eliminate left atrial triggers of atrial fibrillation [7], by recent analyses of ion channel expression and function in left and right atria [8], and by the identification of rare somatic mutations in left atrial myocardium associated with atrial fibrillation [9].

In the last years, several genome-wide association studies in European and Asian populations have confirmed an association between atrial fibrillation and intergenic variations on chromosome 4q25, close to the *Pitx2* transcription factor gene [10], [11]. Heterozygous deletion of *Pitx2c*, the cardiac isoform of *Pitx2*, in mice is sufficient to provoke increased inducibility of atrial fibrillation without obvious structural cardiac alterations [12], [13], associated with a shortening of the left atrial action potential duration [12]. *Pitx2c* has a number of developmental functions, particularly as the final mediator in the left-right patterning pathway of mammalian embryos [14], where it is expressed exclusively on the left, including in the primitive left atrium [15]. Surprisingly, there is also a marked chamber specificity of *Pitx2c* expression in the adult heart: mRNA transcripts are expressed almost 100-fold higher in the left as compared to the right adult human and murine atrium [12].

Taken together, these observations suggest that adult left and right atria differ in their gene expression patterns, and that these differences may generate specific molecular patterns with relevance to atrial pathophysiology. The differences in gene expression between right and left atria have, however, not yet been systematically studied using contemporary genomic techniques. We therefore performed a genome-wide expression analysis in tissues from left and right atria from mice of different genetic backgrounds and age groups, and thereafter confirmed some of the main identified differences in tissue from human atria. We identified *Pitx2c* as the single most enriched gene in left atria of mice and humans, and characterized 76 other atrial genes from different gene groups that are differentially expressed in right and left atria. These findings may help to better target research aimed at identifying new molecular determinants of atrial fibrillation.

## Results

### Genome-wide differences in left and right atrial mRNA expression

**Figure 1:**
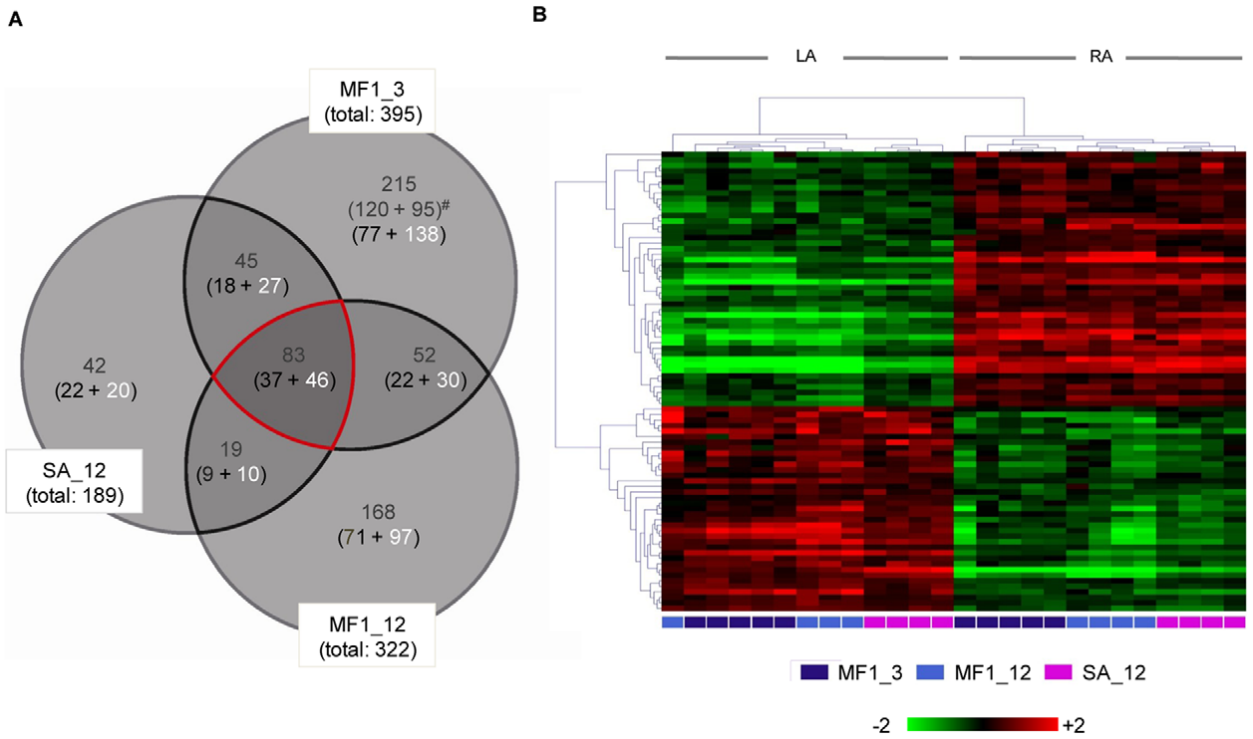
Results of the mRNA expression array experiments. *A* Venn diagram showing the overlap of transcripts (grey numbers) that are significantly different between LA and RA (Fold-Change>1.5, *P*<0.05) in 3-month-old MF1 mice (MF1_3), 12-month-old MF1 mice (MF1-12), and 12-month-old Swiss-Agouti mice (SA_12). The number of transcripts with increased level of expression in the left atrium (black) or right atrium (white) are given in brackets. The overlapping 83 transcripts in the centre of the diagram refer to 77 single genes (see Tables 1 and 2). *B* Two-dimensional hierarchical clustering of relative change in gene expression using the 83 transcripts common to mice of all strains and age groups.

In the 3 microarray datasets (MF1_3, MF1_12, SA_12) a total number of 624 gene probes were found to be significantly differentially expressed in the mouse left and right atrium (Figure 1A, heat map in Figure 1B, complete list in Supplementary Table S1), corresponding to 576 transcripts of 534 genes. Of these, 118 gene probes overlapped between two and 83 probes across all three datasets, corresponding to 77 genes (see Tables 1 and 2). All overlapping genes had consistently higher expression values on the left or right atrial side across all datasets. Overall, more gene probes had higher expression values on the right atrial side (59%, 368 vs. 256). Different array probes targeting the same genes usually gave highly consistent (>95% of cases) results in terms of differential gene expression, suggesting overall reliable data from a technical point of view.

**Table 1:** Genes enriched in left atrium (*P*<0.05) identified in all 3 gene array datasets.

Symbol	Gene Name	Fold change(left vs. right atrium)		
		MF1_3	MF1_12	SA_12
Abcb4	ATP-binding cassette, sub-family B (MDR/TAP), member 4	1.56	1.88**	1.51
Ccl11	Small chemokine (C-C motif) ligand 11	1.80	2.37	3.18
Ccl21b	Chemokine (C-C motif) ligand 21b	8.74*	4.22	2.72
Ccl21c	Chemokine (C-C motif) ligand 21c (leucine)	5.16	3.08	3.04
Ckmt2	Creatine kinase, mitochondrial 2	1.55	1.54	2.30
Cxcl14	Chemokine (C-X-C motif) ligand 14	3.75	2.37	2.54
D630003M21Rik	RIKEN cD D630003M21 gene	2.70	1.72	1.85
Ddit4l	D-damage-inducible transcript 4-like	6.50	3.51	3.89
Entpd2	Ectonucleoside triphosphate diphosphohydrolase 2	1.87	1.80	2.23
F13a1	Coagulation factor XIII, A1 subunit	1.71	2.59	2.08
Fblim1	Filamin binding LIM protein 1	1.54	1.89	1.62
Fcna	Ficolin A	1.79	2.64**	2.14
Gm1631	Gene model 1631, (NCBI)	2.16	1.80	1.54
Gucy1a3	Guanylate cyclase 1, soluble, alpha 3	2.29	2.36	1.86
LOC100041504	Similar to beta chemokine Exodus-2 (Ccl21c)	5.91	2.88	2.74
LOC100048554	Similar to monocyte chemoattractant protein-2 (MCP-2)	2.85	2.96	1.93
Mapk10	Mitogen-activated protein kise 10	2.75	1.92	1.50
Mfap4	Microfibrillar-associated protein 4	2.10	2.47	2.43
Mgl1	Macrophage galactose N-acetyl-galactosamine specific lectin 1	2.61	3.98	1.97
Phlda1	Pleckstrin homology-like domain, family A, member 1	4.57	3.82	5.09
Pi16	Peptidase inhibitor 16	3.20	3.19	2.85
Pitx2	Paired-like homeodomain transcription factor 2	15.43	8.07	10.27
Ppp1r1b	Protein phosphatase 1, regulatory (inhibitor) subunit 1B	4.08	3.65	3.38
Psat1	Phosphoserine aminotransferase 1	1.82	2.69*	1.57
Reln	Reelin	2.39	2.30	2.55
Scara5	Scavenger receptor class A, member 5 (putative)	3.81	4.57	4.13
Sh3gl2	SH3-domain GRB2-like 2	1.87	1.71	2.28
Slc41a3	Solute carrier family 41, member 3	1.74	2.01	1.71
Slco2b1	Solute carrier organic anion transporter family, member 2b1	2.86	2.40	2.17
Syn2	Sypsin II	1.90*	1.84	2.38
Timp4	Tissue inhibitor of metalloproteise 4	1.93	2.77*	2.33
Tnni2	Troponin I, skeletal, fast 2	3.78	2.60	2.33
Uts2d	Urotensin 2 domain containing	2.65	4.99	2.30
Vtn	Vitronectin	2.00	2.14	1.69

Asterisks indicate significant differences between gene expression levels in 3- and 12-months-old MF1 mice (1st column vs. 2nd column; * = *P*<0.05, ** = *P*<0.01).

**Table 2:** Genes enriched in right atrium (*P*<0.05) identified in all 3 gene array datasets.

Symbol	Gene Name	Fold change(left vs. right atrium)		
		MF1_3	MF1_12	SA_12
1500015O10Rik	RIKEN cD 1500015O10 gene	2.06	2.62	2.06
Adm	Adrenomedullin	7.84	7.66	4.02
Ahsg	Alpha-2-HS-glycoprotein	2.18	3.36**	1.72
Aldh1l2	Aldehyde dehydrogese 1 family, member L2	6.23	2.64	1.74
Amigo2	Adhesion molecule with Ig like domain 2	1.98	2.36	2.00
Angptl7	Angiopoietin-like 7	3.13	2.93	1.87
Apoe	Apolipoprotein E	1.51	2.07	1.76
Arc	Activity regulated cytoskeletal-associated protein	1.69	1.70	1.63
Arpp21	Cyclic AMP-regulated phosphoprotein, 21	4.01	2.15	2.47
Asah3l	N-acylsphingosine amidohydrolase 3-like	1.80	2.70*	2.00
BC022687	cD sequence BC022687	2.23	2.62	2.05
Bmp10	Bone morphogenetic protein 10	27.04*	4.23	12.55
Cd163	CD 163 antigen	1.52	1.66**	1.58
Cd207	CD 207 antigen	16.46	18.77**	10.72
Ckmt1	Creatine kinase, mitochondrial 1, ubiquitous	1.59	1.81	1.59
Cxcl13	Chemokine (C-X-C motif) ligand 13	13.98	14.52*	7.59
Cyp1b1	Cytochrome P450, family 1, subfamily b, polypeptide 1	1.61	1.85	1.54
Dbh	Dopamine beta hydroxylase	4.04	5.39	2.86
Dok4	Docking protein 4	1.96	1.67	1.62
Ecm1	Extracellular matrix protein 1	1.78	1.86**	1.51
Emilin2	Elastin microfibril interfacer 2	2.82	2.74	2.24
Fxyd3	FXYD domain-containing ion transport regulator 3	4.97**	2.79	3.13
Gng13	Guanine nucleotide binding protein 13, gamma	3.41	3.14	2.60
Hamp	Hepcidin antimicrobial peptide	10.78	17.15	7.00
Hamp2	Hepcidin antimicrobial peptide 2	9.05	12.89	9.21
Hdc	Histidine decarboxylase	1.85	2.06	1.96
Hey1	Hairy/enhancer-of-split related with YRPW motif 1	2.50	2.55	2.24
Id1	Inhibitor of D binding 1	2.29*	1.83	1.80
Id2	Inhibitor of D binding 2	1.91	2.87	1.94
Id3	Inhibitor of D binding 3	1.54	1.84	1.69
Igfbp3	Insulin-like growth factor binding protein 3	2.60	2.15	3.81
Klk8	Kallikrein related-peptidase 8	1.59	1.60	1.65
LOC670044	Similar to Mothers against decapentaplegic homolog 6	2.61	2.99	2.67
Mrvi1	MRV integration site 1	1.57	1.70**	1.55
Msc	Musculin	3.18	2.51	2.41
Pla2g5	Phospholipase A2, group V	1.98	1.65	1.65
Ptgds	Prostaglandin D2 synthase	4.50	5.15	3.00
Ryr3	Rryanodine receptor 3	2.87	3.03**	2.72
Smarcd3	regulator of chromatin, subfamily d, member 3	1.92	2.08*	2.07
Tcf21	Transcription factor 21	1.87	1.95	1.97
Tmem108	Transmembrane protein 108	3.14	3.90	2.44
Vsig4	V-set and immunoglobulin domain containing 4	16.72	5.83	7.81
Vwf	Von Willebrand factor homolog	1.79	2.03**	1.70

Asterisks indicate significant differences between gene expression levels in 3- and 12-months-old MF1 mice (1st column vs. 2nd column; * = *P*<0.05, ** = *P*<0.01).

### Gene Set Enrichment Analysis

To investigate the potential relevance of differentially expressed genes in left and right atrium without preformed hypotheses, we performed gene set enrichment analyses (GSEA) in each of the datasets independently. We identified a total of 119 differentially expressed Gene Ontology (GO) terms, with the majority of these enriched in right atrium (77 terms) and in the MF1_3 dataset (107 terms, Supplementary Table S2). Of those we found to be enriched in left atrium, 10 were mitochondria-related GO terms (of which 3 were identified across datasets), ribosome-related terms, and protein kinase regulation terms. Of those with right atrial predominance, we found enriched GO terms relating to transforming growth factor-β (TGFβ) signalling, humoral processes, steroid biosynthesis, immune response, muscle development, extracellular matrix composition, and transmembrane receptor activity.

### Single Gene Enrichment Analysis

**Figure 2:**
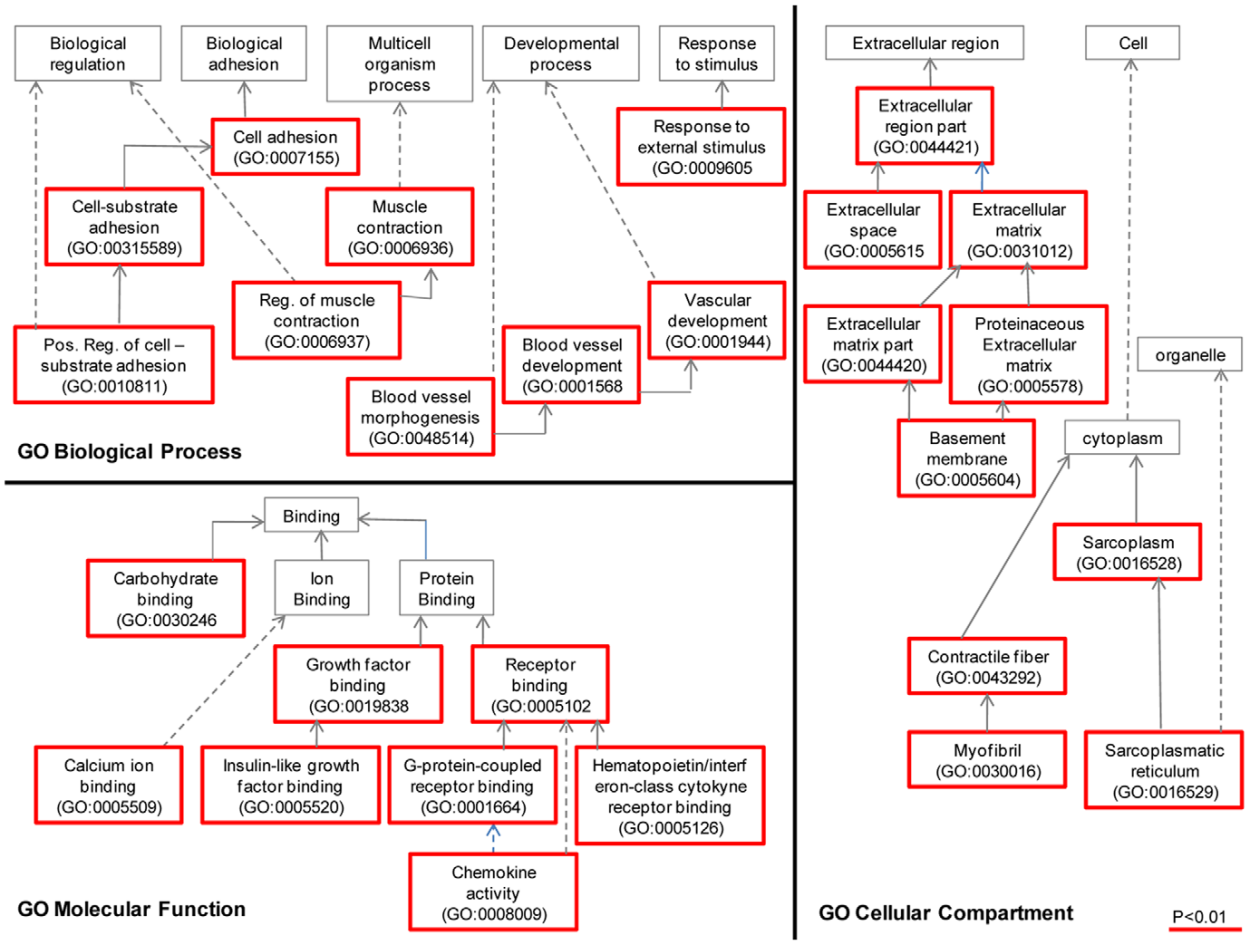
Gene groups enriched in left atrium according to FatiGO gene ontology analysis. 220 genes significantly enriched in the left atrium in any of the three microarray datasets (MF1_3; MF1_12; SA_12) were used as input list against the genome. 9 biological process, 10 cellular component, and 8 molecular function enriched GO classes are highlighted in red (levels 3–9; P<0.01).

To further evaluate how the preferential expression pattern in the left atrium might affect left atrial function, we performed Gene Ontology analysis using FatiGO. All 220 genes enriched in left atrium by array analysis were recognized by the software that could assign GO-biological process terms to 146 genes, GO-cellular component terms to 115, and GO-molecular function to 155. Using the significantly enriched genes in left atrium as input list, 9 GO-biological process terms (levels 3–9), 10 GO-cellular component terms, and 8 GO-molecular function terms were significantly enriched (*P*<0.01, Figure 2, for a complete list of GO terms see Supplementary Table S3).

In both left and right atrium most of the specifically expressed genes are coding for proteins expressed in the extracellular region (extracellular matrix and space), as well as for contractile fibers and myofibrils. The biological process analysis revealed in both atria an over-representation of GO categories related to blood vessels development and morphogenesis, response to external stimuli, and cell adhesion. In the left atrium, we observed an enrichment of genes involved in the regulation of muscle contraction. In addition, we found groups of genes involved in immune and inflammatory responses, cell differentiation and proliferation, and regulation of transcription and apoptosis. In both atria, molecular function terms specific for binding of growth factors, receptors, carbohydrates, and calcium were enriched. In the left atrium, chemokine activity and cytokine receptor binding were also enriched.

Overall (see below), the pathway analyses confirm our main observations of individual gene expression differences in murine atrial tissue, and are consistent with our validation experiments in human atrial tissues.

### Validation of array-based gene expression profiles by RT-qPCR in mouse tissues

**Figure 3:**
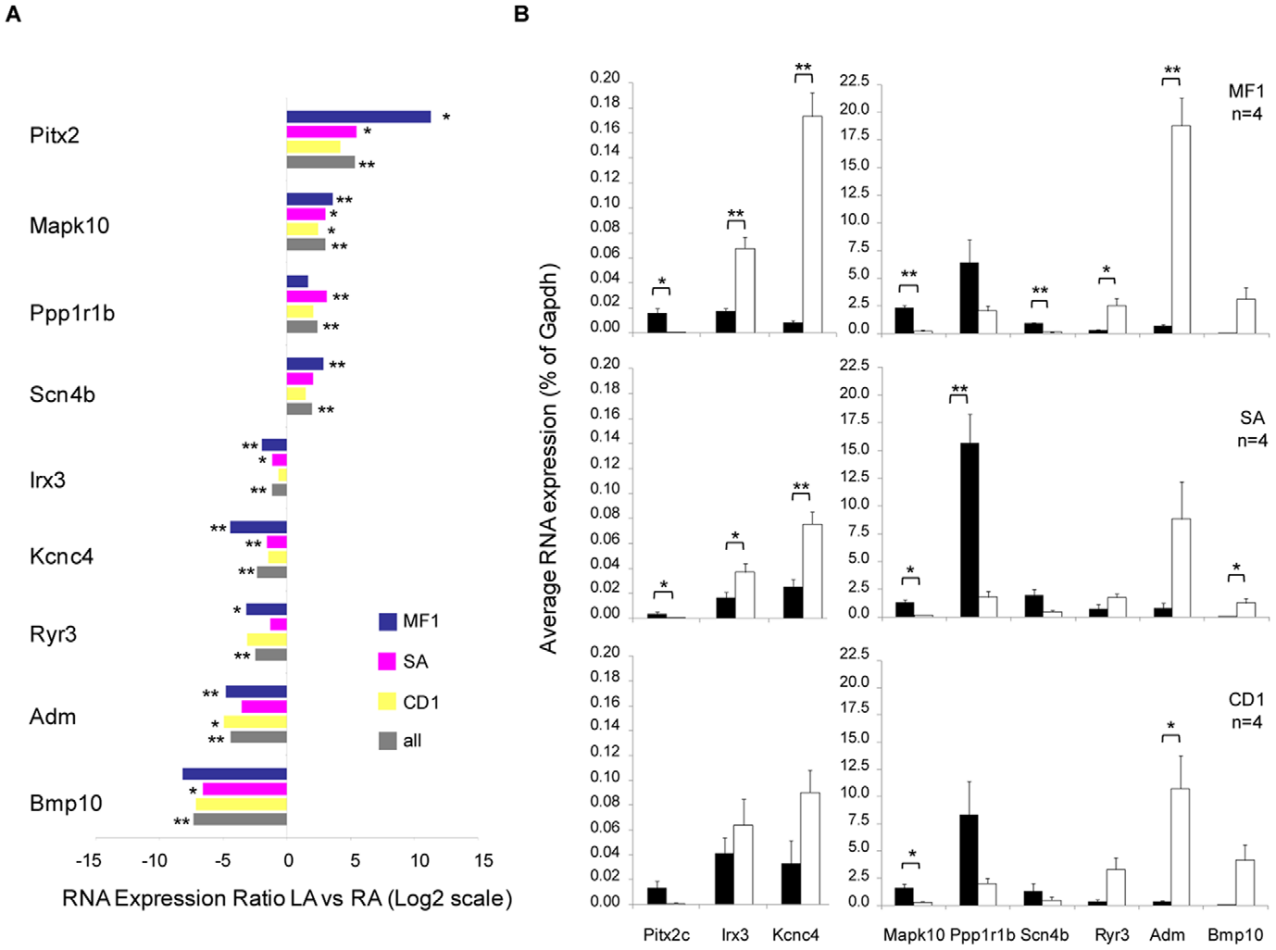
RT-qPCR validation of the differential expression of candidate genes in left and right atria from wild-type mice of three different strains. *A* Relative Log2 expression ratios between LA and RA in MF1 mice (blue, n = 4), Swiss-Agouti mice (pink, n = 4), CD1 mice (yellow, n = 4), and in wild-type mice (grey, n = 12). *B* Corresponding average mRNA expression in left (black) and right (white) atrial tissues of MF1, SA, and CD1 mice (n = 4 each). *Gapdh* was used as control. Error bars indicate standard error of the mean (SEM). Statistically significant differences as assessed by unpaired Student's t-test are represented by asterisks (*P<0.05, **P<0.01).

To validate the microarray data set, we selected left (n = 5) and right (n = 4) marker candidates with different degrees of overlap between the array datasets (2 genes identified in only 1 dataset, 1 gene in 2 datasets, and 6 genes identified in 3 datasets). We assessed mRNA concentrations by RT-qPCR on additional samples of MF1 mice (n = 4, 3-month-old) and Swiss-Agouti (n = 4, 12-months-old), and on a third wild-type mouse strain (CD1, n = 4, 3.5-months-old). All genes tested by RT-qPCR were significantly differently expressed between left and right atrial tissues in at least one of the wild-type mouse strains (Figure 3A–B). For all genes, the atrium showing enriched expression was the same by array and RT-qPCR. Overall, RT-qPCR confirmed the microarray data, both in enrichment in a given tissue (right versus left atrium) and across different mouse strains (Figure 3A and 3B).

### Age-dependent differences

Our array experiments were designed to identify differences in gene expression that are independent of the strain and the age of the mouse. The data set also allowed for an exploratory analysis of age-dependent differences in gene expression in mouse atria of MF_1 mice. This analysis identified 140 genes that were upregulated by at least 2-fold (*P*<0.05) in the left atrium of 12-months–old MF_1 mice, and 268 upregulated genes in left atrium of 3-month–old. Of the 77 genes listed in Tables 1 and 2, four genes showed enhanced expression in the left atrium of the older mice and only 2 in left atrium of the younger mice. Ten genes were enriched in the right atrium of the older mice and only 3 in the right atrium of the younger mice. These genes are marked by asterisks in Tables 1 and 2. Comparison of MF1 gene arrays in the 3- and 12-month–old mice revealed no significant difference in *Pitx2c* expression. Expression of *Irx3* was significantly downregulated in the right atrium of the 12-month–old mice compared with the 3-month–old mice, whereas expression of *Scn4b* was upregulated with age (data not shown).

### Validation of differential gene and protein levels in human atrial tissues

**Figure 4:**
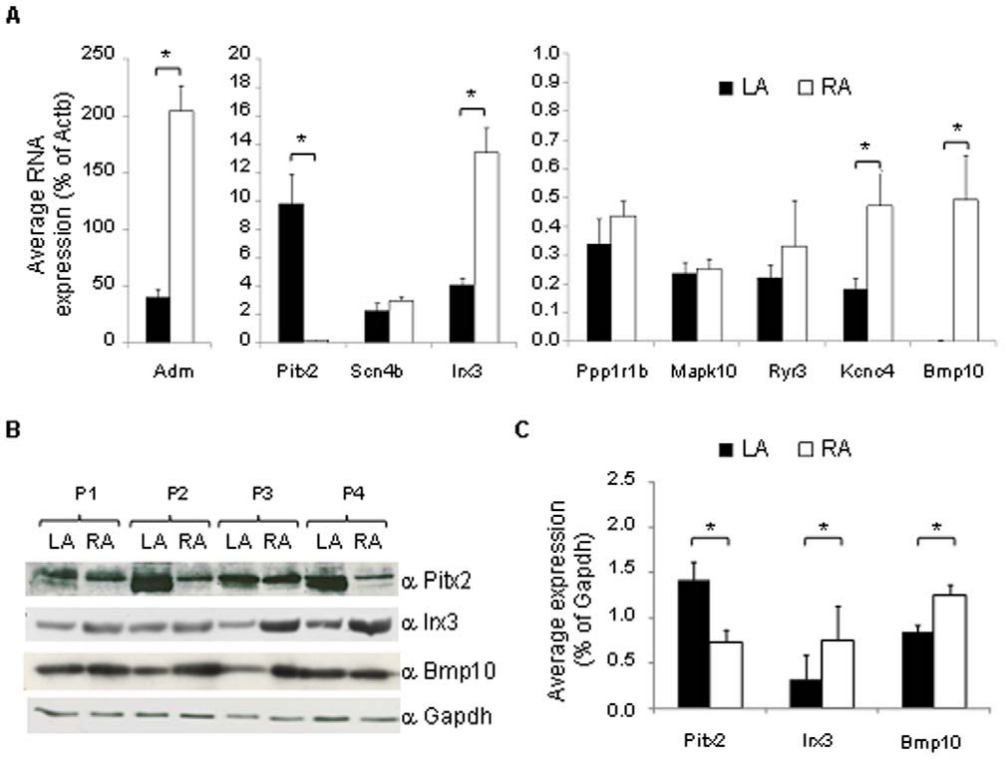
A) RT-qPCR measurements of orthologous transcripts in left atrial (black) and right atrial (white) human appendages (n = 5). *Actb* was used as control. Error bars indicate standard error of the mean (SEM). Statistically significant differences as assessed by paired Student's t-test are represented by asterisks (*P<0.05). B) and C) PITX2, IRX3, and BMP10 protein expression level measured by Western blot of total protein lysates from human left (black) and right (white) atrial appendages (n = 4). The data shown are averages of the results obtained from four separate samples. Error bars indicate standard deviation (SD). Statistically significant differences as assessed by unpaired Student's t-test are represented by asterisks (*P<0.05).

To determine whether the differences identified in the left and right atrial gene expression in the mouse are consistent in humans, we performed RT-qPCR measurements on human left and right atrial appendages (n = 5) harvested from patients undergoing open heart surgery. Clinical characteristics are given in Table 3. The RT-qPCR measurements confirmed differential expression in the left and right atrium in 5 of 9 tested human orthologues (Fig. 4A). *Adm*, *Irx3*, *Kcn4*, and *Bmp10* were confirmed to be expressed at higher levels in right than in left atrium, whereas *Pitx2c* was enriched in left atrium, as expected. The murine left atrium-enriched genes *Mapk10*, *Ppp1r1b*, and *Scn4b*, and *Ryr3*, enriched in right atrium by array analysis, were not confirmed as such in the human tissue samples used for analysis.

**Table 3:** Experimental setup and samples.

	Murine atria				Human atria				
	MF1	MF1	Swiss Agouti	CD1	P1	P2	P3	P4	P5
**Age**	12±0 w	52±0 w	52±0 w	14±0 w	55 y	60 y	82 y	86 y	64 y
**Sex**	9 f	4 m	8 f	2 m+2 f	f	m	f	m	m
**Operation**	-	-	-	-	AVR	CABG	AVR	AVR CABG	CABG
**CHADS2 score** #	-	-	-	-	0	2	2	3	2
**Array**	x	x	x	-	-	-	-	-	-
**RT-qPCR**	x	-	x	x	x	x	x	x	x
**Western-Blot**	-	-	-	-	x	x	x	x	-

Information on the types of samples used for the different experimental protocols. For human samples (P1–P5), clinical characteristics are given. All patients were in permanent atrial fibrillation at the time of tissue harvesting during open heart procedures (AVR = Aortic Valve Replacement; CABG = Coronary Artery Bypass Grafting). All sample pairs (left and right atrium) were preserved under standardized, pairwise identical conditions as described in the text.

# The CHADS2 is a clinically used score that summarises comorbidities often found in atrial fibrillation patients.

It adds one point each for heart failure, hypertension, diabetes mellitus, age >75 years, and two points for a prior stroke.

Furthermore, we confirmed higher expression of three selected genes at the protein level. Using Western blots of lysates prepared from immediately shock-frozen human left and right atrial appendages from the same human samples, we confirmed higher PITX2 protein levels in left atrium and higher IRX3 and BMP10 protein levels in right atrium (n = 4, Fig. 4B and C).

## Discussion

### Main findings

This systematic analysis of gene expression profiles identified major differences in gene expression between left and right adult atrial tissue. These observations were not only consistent across different strains of murine hearts, but also confirmed in human atria. These data shed light onto potentially relevant physiological differences between right and left atria: Our hypothesis-free analysis identified *Pitx2c*, a gene that has recently been shown to be involved in the pathophysiology of atrial fibrillation, as the single most enriched gene in the left atrium, while *Bmp10*, a gene known to be relevant for trabeculation of the right atrium and ventricle, was the single most differentially expressed gene in the right atrium. Other differentially expressed genes are involved in membrane electrophysiology, metabolic cellular function, and regulation of inflammatory cells.

### Significant differences in left and right atrial gene expression

We systematically characterized gene expression differences between the right and left atria by gene arrays in healthy mice of different strains and at different ages, and by employing different commercially available gene array analyses in two different laboratories. The degree of consistency in left-/rightness among probes that were identified in murine atria from mice of different strains and ages, as well as the number of transcripts that were identified by multiple probes, highlight the robustness of the observed differences. This finding is affirmed by the unbiased detection of overlapping GO terms by GSEA and FatiGO in the different datasets (Supplementary Tables S2 and S3). Although we have highest confidence in those genes that were identified in all 3 datasets, we assume that some non-overlapping genes may represent true biological differences. Finally, differences in individual genes were validated by RT-qPCR in murine tissue. Several differences were furthermore validated on human atrial tissue at the RNA and protein levels.

### What controls “sidedness”?

Given that the constituent cells types of the left and right atria do not differ markedly, the consistent differential gene expression raises the question of how this is controlled. By extension of its role in embryonic patterning, *Pitx2c* is one obvious candidate for a controlling transcription factor. The embryonic right-side developmental programme has been considered as the “default” pathway, which will be followed unless signals are received instructing otherwise. For heart development, *Pitx2c* is the key mediator for such signalling, and in its complete absence, “leftness” is lost and right isomerism results [16]. Our finding that *Pitx2c* is the most highly differentially expressed gene in adult left atria is compatible with a continuing control function. Only a few genes have been identified as direct targets of *Pitx2*, including *Cyclin D2 (Ccnd2)* and *Lef-1*, but none of the differentially expressed atrial genes we have identified have been studied in this regard. Interestingly, our most highly expressed right atrial gene, *Bmp10*, has been reported to be regulated by *Pitx2*c, although a direct effect has not been tested [17]. In that case, as well as for some sinuatrial node genes [12], [13], the effect of *Pitx2c* is to repress expression in the left atrium. *Bmp10* is best known for its role in cardiomyocyte growth in trabecular and compact myocardium [18], [19]. As we found canonical target genes of the BMP signaling pathway enriched in right atrium (*Id1*, *Id2*, *Id3*) in our experiments and in a prior analysis of pooled murine atria using self-constructed gene arrays [20], it may be speculated that there is an “active” growth regulatory role for *Bmp10* in the adult right atrium. Alternatively, Bmp10 could have a reciprocal role to *Pitx2c* in maintaining left-right atrial differences by suppressing genes in the right atrium. In support of this notion, BMP signalling is known to have a role in suppressing the nodal-pitx2 pathway in early embryonic left-right patterning [21]. One approach to identify genes that are regulated in the atria by *Pitx2c* would be correlation analysis using microarrays, looking for genes that are co-regulated with *Pitx2c* or with *Bmp10* [22].

### *Pitx2c* is the single most differentially expressed gene between the left and right atrium

*Pitx2c* emerged as the single most differentially expressed gene between left and right atrial tissue. This finding was consistent well into advanced adulthood (12 months of age). Higher left than right atrial expression of *PITX2c* was confirmed in human left atria. Of the seven differentially expressed genes with a role in cardiac development and morphogenesis, six are highly expressed in the right atrium (*Adm*, *Bmp10*, *Id1*, *Id2*, *Id3*, *Smarcd3*) and only *Pitx2c* is highly expressed in the left atrium. This differential expression is conserved between mice and men, and results in a left-right PITX2 protein gradient (Figure 4). This new observation substantiates our prior assumption that *Pitx2c* has a functional role in the adult left atrium [12], and that loss of *Pitx2c* function may revert left atrial cardiomyocytes to right-sidedness.

### Reduced *Pitx2c* expression reduces “leftness” of the genomic signature in murine atria

To assess to what extent *Pitx2c* might control leftness in the healthy murine atrium, we compared the 10 most highly differentially expressed left and right atrial genes with a previously published dataset of *Pitx2c^+/−^* mice, that were littermates to the MF1_3 animals used in this present study [12]. Of the genes with highest left-right fold-change (excluding *Pitx2c*), 5 of 9 were reduced to 50–60% of wild-type levels in *Pitx2c^+/−^* left atria (Table 4; *Ccl21b*, *Ddit4l*, *LOC100041504*, *Ccl21c*, and *Ppp1r1b*). This suggests that these genes are positively induced by *Pitx2c*; the remaining 4 genes do not appear to be controlled by *Pitx2c*. Similarly, among the 10 genes most enriched genes in right atrium, 4 were increased by 140–520% in the left atrium of *Pitx2c^+/−^* (Table 4; *Bmp10, Vsig4, Cd207, and Cxcl13*), suggesting that these genes are repressed by *Pitx2c*. We cannot say whether these apparent inductions and repressions are direct or indirect. Nevertheless, this comparison suggests that about half of the differences in gene expression seen in the left versus right atria may be secondary to regulation by *Pitx2c*. Maintaining atrial “left-sidedness” through adequate *Pitx2c* expression could even be a regulatory pathway that could help protect left atria against development of atrial fibrillation [10], [12], [13].

**Table 4:** Top 10 genes enriched in left atrium in MF1_3 dataset (excluding *Pitx2c*) compared to gene expression in left atrium of 3-month–old *Pitx2c^+/−^* mice [12].

	MF1_3	*Pitx2c^+/−^* vs. MF1_3
Gene	FC (LA vs. RA)	(LA)
Ccl21b	8.74	53.0%
Ddit4l	6.50	64.8%
LOC100041504	5.91	60.4%
Ccl21c	5.16	61.0%
Phlda1	4.57	101.7%
Ppp1r1b	4.08	58.5%
Scara5	3.81	114.5%
Tnni2	3.78	85.6%
Cxcl14	3.75	79.3%

### Gene expression differences in ion channel composition

Several reports have consistently shown that left atrial action potential duration (APD) is shorter than right atrial APD in mice [23] and in canines [24]. The RNA for I_K,ACh_, *Kcnc4*, was consistently expressed at higher levels in right atria in mice and humans. Hence, a “loss of left-sidedness” would increase I_K,ACh_ levels in the left atrium, which may contribute to atrial fibrillation [25], [26].

### Cytokine 21 ligand genes are highly expressed in the left atrium

Genes coding for cytokines and cytokines receptors are found in both atria (left atrium: *Ccl11*, *L0c100041504*, *Cxcl14*, *Ccl21c*, *Ccl21b*, *Vtn*; right atrium: *Dbh*, *Ahsg*, *Cd163 Cxcl13*). Of note, 3 transcripts encoding the cytokine 21 ligand (isoforms b and c) were robustly and highly enriched in left atrium. It is tempting to speculate that this cytokine receptor, which regulates chemotaxis and formation of lymph nodes, may be involved in the production of myeloperoxidase in the left atrium, which has recently been linked to development of atrial fibrillation and atrial fibrosis [27]. Further studies on the role for *Ccl21* in left atrial function are warranted. Consistent with this, we find other genes involved in inflammatory processes with higher expression in the left atrium: *Timp4* was differentially expressed in all three data sets (Table 1), and *Alox5*, *C3*, and *Timp3* were differentially expressed in some, but not all tissues examined (Supplementary Table S1). The role of local inflammation in the pathogenesis of atrial fibrillation has long been under discussion [28], [29], and anti-inflammatory agents can prevent post-operative atrial fibrillation [3]. Recently, alterations in the expression of *Timp2* and *Mmp2* have been shown to be associated with atrial fibrillation in different respects [30], [31], with the latter enriched in left atrium in two out of three of our datasets (Supplementary Table S1).

### Other genes enriched in the left atrium

Among the 77 genes enriched in the left or right atrium in all three array experiments, 2 genes with a left-sided enrichment have a role in the blood coagulation (*F13a1*, *Entpd2*). Four left-sided genes (*Fblim1*, *Reln*, *Vtn*, *Mfap4*) and 3 right-sided genes (*Emilin2*, *Amigo2*, *Vwf*) code for adhesion proteins. *Tcf21*, *Hey1*, *Id1*, *Apoe*, *Dbh* (right atrium), and *Pitx2c* (left atrium) are involved in blood vessel development. Left atrial clot formation is the basis for thrombembolic stroke, one of the severe complications of atrial fibrillation. In our dataset we also found genes related to coagulation, platelet activity and thrombogenesis as being left-right differentially expressed in the atria (left atrium: *Entpd2*, *F13a1*, *Vtn*; right atrium: *Vwf*, *Mrvi1*, *Cxcl14*). Recently, *Cxcl14*, also known as Pf4 (platelet factor 4), was found to be slightly elevated in the left atrium of patients with atrial fibrillation [32]. *Ddit4l*, another highly differentially expressed gene, codes for a protein involved in DNA damage and hypoxia-induced cell death. *Ppp1r1b*, another gene with preferential left atrial expression, codes for a regulatory subunit of protein phosphatase 1 (PP1). The catalytic subunit of PP1 and its inhibitor are implicated in the development of heart failure at the ventricular level [33], [34], [35]. The relevance of the regulatory subunit encoded by *Ppp1r1b* in the atrial myocardium has not been studied.

### Implications

Our study identifies relevant differences in gene expression between left and right atrium, suggesting that analysis of genes and proteins should be separated for each of the two chambers when functionally relevant differences are at stake. Our study also confirms that *Pitx2c* is highly expressed in the left atrium, suggesting a relevant role for this gene for left atrial function in health and disease. Combined with the published information that reduced *Pitx2c* expression is a predisposing factor for atrial fibrillation [10], [12], [13], our data suggest that maintaining “left-sidedness” in the left atrium may be important for normal left atrial function in the adult heart.

### Limitations

Here, we identify relevant differences in gene expression between the right and left atria based on modern, relatively comprehensive gene array technology. While we were able to confirm some of our findings by RT-qPCR, including analyses of diseased human tissues, our study will certainly miss further, relevant biological differences between the right and left atria that may be picked up by more sensitive, hypothesis-driven analyses. This is a shortcoming generic to array analyses. The hypothesis-free analysis of atrial tissue was confined to murine datasets in our study. Our validation of the differences in the expression of some murine genes in human atrial tissue harvested from patients with heart disease requiring open heart surgery suggests that gene expression differences may have biological relevance to human atrial function. Further studies are warranted to study the effects of heart disease on differential gene expression in the left and right atrium. Furthermore, not all differences in mRNA concentrations translate into differences of protein concentration [36].

## Methods

### Murine atrial tissues

We studied wild-type mice of three different outbred strains that are commonly used in research laboratories (MF1, Swiss-Agouti, and CD1). Animals were kept and sacrificed in one facility in Münster, Germany, following identical tissue extraction protocols. The harvesting of animal tissues occurred within a research program approved by our local authority (lanuv NRW). For array analyses, we used 3-months-old (12±0 weeks) and 12-months-old (52±0 weeks) MF1 mice and 3-months-old (12±0 weeks) Swiss-Agouti mice. For confirmatory RT-PCR experiments, we additionally analyzed cardiac tissue from 3-month-old (14±0 weeks) CD1 mice. Hearts were dissected in cold buffer solution and left and right atria were stored separately. Tissues from 3-month-old MF1 mice were placed into RNAlater (QIAGEN, Hilden, Germany) and shipped to the second laboratory in London, United Kingdom, for microarray experiments. All other samples were shock-frozen in liquid nitrogen immediately after preparation and kept at −80°C until further processing. Preparation time from the incision of the murine thorax to preservation of the tissue was less than 3 minutes (172±21 seconds).

### Human atrial tissue

Human samples from left and right atrial appendages were collected during open heart surgery, shock-frozen and stored at −80°C. All patients gave written informed consent to the preservation and analysis of their tissue, and the analysis was approved by the ethics committee of Ärztekammer Westfalen-Lippe and Medical Faculty of WWU Münster (AZ 2006-414-f-M).

### Tissue homogenization and RNA isolation

All collected samples were handled independently and individually throughout the experiments, with no pooling of any samples, at difference to prior reports using custom-made, non standardized arrays [20]. Collected tissues were pulverised at 3000 rpm for 45 sec using a Mikro-Dismembrator S (Sartorius, Goettingen, Germany). Half of the material was used for extraction of total RNA, the other half for protein isolations. Total RNA was extracted following standard techniques using RNeasy Fibrous Tissue Mini Kit and Micro Kit (QIAGEN; including on-column DNAse treatment) from human and mouse samples. Isolated RNA was quantified by UV spectrophotometry and checked for integrity by capillary electrophoresis using an Agilent 2100 Bioanalyzer (Agilent technologies, Boeblingen, Germany). RNA Integrity Number >8 was accepted for further experiments.

### Mircoarray hybridization

We performed 3 separate microarray experiments comparing left and right atrial gene expression: *“MF1_3”* using 3-month-old MF1 mice (n = 5, Illumina MouseWG-6 v2.0 Expression BeadChip, 45,281 probes), *“MF1_12”* on 12-month-old MF1 mice (n = 4, Illumina MouseRef-8 v2.0 Expression BeadChip, 25,697 probes) and “SA_12”on 12-month-old Swiss-Agouti mice (n = 4, Illumina MouseRef-8 v2.0 Expression BeadChip). For each experiment, 100 ng of total RNA was labelled using the Illumina Total Prep RNA Amplification Kit (Ambion, Austin, TX, USA) and then hybridized to the chips. Scanning was performed on a Bead Array Reader (Illumina, Eindhoven, The Netherlands) and data was extracted from the images with Genome Studio (Version 2009.1, Illumina). All chips passed quality control to eliminate scans with abnormal characteristics. Expression data was deposited at the Gene Expression Omnibus (GEO accession numbers: GSE22170, GSE29500).

### Quantitative Real-Time Polymerase Chain Reaction

We used quantitative real-time polymerase chain reaction (RT-qPCR) to validate the levels of expression from the microarrays. Up to 1 µg of RNA per sample was reverse transcribed into cDNA using oligo-dT_15_ primers and M-MLV Reverse Transcriptase (USB) following standard procedures. All primers were designed with Primer3 software [37] and validated with respect to efficiency and specificity. Experiments were carried out using Power SYBR Green PCR Master Mix (Applied Biosystems). All measurements were performed in duplicate. Relative quantification of expression was performed according to the “ΔΔCt method” [38] and by employing Glyceraldehyde-3-Phosphate Dehydrogenase (*Gapdh*) or Beta-Actin (*Actb*) for normalization. (For full details on the primer sequences and RT-qPCR settings, see Supplementary Table S4.)

### Protein biochemistry

Pulverized human left and right atrial samples were resuspensed in lysis buffer (50 mM Tris-Cl, pH 7.4, 150 mM NaCl, 0.5% Triton X-100), supplemented with a protease inhibitor cocktail tablet (Complete Mini, Roche diagnostics, Mannheim, Germany). Homogenates were then centrifuged at 2500 rpm for 5 minutes. 50 µg of total protein lysate was loaded onto a 10% SDS-polyacrylamide resolving gel (1-mm thick) and electrophoresed for 75 minutes at 180 V. Gels were blotted onto a nitrocellulose membrane (Hybond ECL, GE Healthcare, München, Germany) by means of a wet transfer system for 60 minutes at 100 V. The membranes were incubated overnight at 4°C with isoform-unspecific rabbit-derived anti-PITX2 antibody (Capra Science, # PA 1020-100 , 1∶1,000 in blocking buffer), anti-IRX3 (Santa Cruz Biotechnology, # sc-30157, 1∶500 in blocking buffer), mouse-derived anti-BMP10 (R&D Systems, #MAB2926, 1∶500 in blocking buffer [39]) and mouse-derived anti-GAPDH (Ambion, #AM4300, 1∶10,000 in blocking buffer). For better detection of the mature, secreted form of BMP10 without its precursors, membranes used for the human BMP10 blot were cut at 46 kD. After washing, membranes were incubated with anti-rabbit or anti-mouse IgG, HRP-Linked Whole Antibody (1∶10,000; GE Healthcare) for 2 hours at room temperature. Detection was performed using ECL Western blot substrate (Thermo Fisher Scientific, Waltham, MA, USA). Band densities were quantified using Image J (National Institutes of Health, Bethesda, MD, USA).

### Statistical analysis

All 3 microarray datasets (MF1_3, MF1_12, SA_12) were analysed independently. In each dataset, samples were normalized with Genespring (version GX11.0, Agilent technologies) by applying a quantile algorithm and a baseline transformation to the median of all samples. Gene probes with a fold-change >1.5 and a Benjamini-Hochberg corrected *P*-value<0.05 were accepted as significantly different between left and right atria. Two-dimensional hierarchical clustering was performed in MultiExperiment Viewer (TM4 Microarray Software Suite; [40]) on gene probes that were identified as significantly different in all 3 datasets. Unpaired Student's t-test was employed to test for significant differences in relative RNA expression values, as determined by RT-qPCR, and protein expression levels, from Western blot experiments, between the left and right atrium. A two-sided *P*-value<0.05 was accepted as significant.

### Microarray functional analysis

Two independent and unbiased strategies were utilized to identify enriched functional themes (GO terms) within the datasets. Gene Set Enrichment Analysis (GSEA, Broad Institute; standard settings, [41]) was performed on the unfiltered datasets. As suggested for GSEA, a false discovery rate <0.25 and a nominal *P*-value<0.05 were accepted as significant. To assign biological meaning to the group of genes significantly upregulated in the left or right atrium in any of the three datasets (MF1_3; SA_12; MF1_12), the FatiGO algorithm (Babelomics 4.2, corrected p-value<0.01) was applied to compare these lists with the rest of the genome. Additionally, we performed a common normalization for the MF1_3 and MF1_12 datasets to determine whether there were any age-dependent differences in this strain.

## Supporting Information

Table S1 List of 624 gene probes that were identified in any of the three independently prepared mRNA expression arrays comparing left and right atrial gene expression in adult mice. Gene probes that were not assigned in the “smaller” Illumina MouseRef-8 v2 Expression BeadChip, which were used to generate the MF1_12 and SA_12 datasets (compared with the Mouse WG-6 v2 Expression BeadChip for the MF1_3 dataset) are labelled as “na” in the table. If a gene probe did not exhibit a significant difference between left and right atrium in one dataset, the *P*-value column was left blank in this dataset.
(DOCX)

Table S2 Gene set enrichment analysis results.
(DOCX)

Table S3 FaTiGo results (Biological Process; Molecular Function, Cellular Compartment).
(DOCX)

Table S4 RT-qPCR primers for selected murine and orthologous human genes.
(DOCX)
