# Supplementary material for: Systematic Analysis of Gene Expression Differences between Left and Right Atria in Different Mouse Strains and in Human Atrial Tissue
Source: PLoS One. 2011 Oct 19;6(10):e26389. doi: 10.1371/journal.pone.0026389 (PMC3198471; doi:10.1371/journal.pone.0026389)
Supplement: Table S1 — List of 624 gene probes that were identified in any of the three independently prepared mRNA expression arrays comparing left and right atrial gene expression in adult mice. Gene probes that were not assigned in the “smaller” Illumina MouseRef-8 v2 Expression BeadChip, which were used to generate the MF1_12 and SA_12 datasets (compared with the Mouse WG-6 v2 Expression BeadChip for the MF1_3 dataset) are labelled as “na” in the table. If a gene probe did not exhibit a significant difference between left and right atrium in one dataset, the P-value column was left blank in this dataset. (DOCX) [file pone.0026389.s001.docx]

**Supplementary Table S1.** List of 624 gene probes that were identified in any of the three independently prepared mRNA expression arrays comparing left and right atrial gene expression in adult mice.

|  |  |  | MF1_3 |  |  | MF1_12 |  |  | SA_12 |  |  |
| --- | --- | --- | --- | --- | --- | --- | --- | --- | --- | --- | --- |
| Illumina Probe ID | Gene Symbol | Transcript ID | highest | FC (LA vs. RA) | corrected *P*-value | highest | FC (LA vs. RA) | corrected *P*-value | highest | FC (LA vs. RA) | corrected *P*-value |
| 1110435 | 1110007M04Rik | NM_026742.3 | LA | 1.12 |  | LA | 1.50 | 0.011 | LA | 1.26 |  |
| 670356 | 1200009I06Rik | NM_028807.1 | LA | 1.52 | 0.001 | LA | 1.60 | 0.010 | LA | 1.21 |  |
| 540138 | 1500012F01Rik | NM_001081005.1 | RA | 0.98 |  | RA | 0.62 | 0.020 | RA | 0.96 |  |
| 4610504 | 1500015O10Rik | NM_024283.2 | RA | 0.49 | 0.001 | RA | 0.38 | 0.009 | RA | 0.49 | 0.000 |
| 2850112 | 1700025G04Rik | NM_197990.2 | LA | 1.43 |  | LA | 1.54 | 0.015 | LA | 1.34 |  |
| 3120142 | 1700113I22Rik | NM_026865.2 | LA | 1.35 |  | LA | 1.79 | 0.009 | LA | 1.53 | 0.021 |
| 5960097 | 1700113I22Rik | NM_026865.2 | LA | 1.69 | 0.000 | LA | 2.17 | 0.001 | LA | 1.41 |  |
| 1710594 | 1810005K13Rik |  | LA | 1.75 | 0.000 | na | na | na | na | na | na |
| 990221 | 2300002D11Rik | NM_001081156.1 | RA | 0.71 |  | RA | 0.56 | 0.030 | RA | 0.84 |  |
| 5290315 | 2310003M01Rik | | LA | 4.07 | 0.001 | na | na | na | na | na | na |
| 5820725 | 2310015B20Rik | XM_907184.2 | LA | 3.00 | 0.000 | na | na | na | na | na | na |
| 540615 | 2310039E09Rik | NM_026509.1 | RA | 0.94 |  | LA | 1.79 | 0.004 | LA | 1.35 |  |
| 110333 | 2310039L15Rik | XM_290098.1 | LA | 1.72 | 0.000 | na | na | na | na | na | na |
| 7610239 | 2510009E07Rik | NM_001001881.1 | RA | 0.65 | 0.000 | RA | 0.51 | 0.001 | RA | 0.74 |  |
| 3610239 | 2810003C17Rik | NM_145144.1 | RA | 0.47 | 0.001 | RA | 0.57 | 0.011 | RA | 0.65 |  |
| 7200719 | 2810046M22Rik | NM_026621.1 | LA | 1.60 | 0.002 | LA | 1.18 |  | LA | 1.19 |  |
| 5050711 | 2900073G15Rik | NM_026064.1 | RA | 0.92 |  | LA | 1.62 | 0.010 | RA | 0.97 |  |
| 4390195 | 3110006E14Rik | XM_906878.3 | LA | 1.51 | 0.000 | na | na | na | na | na | na |
| 10592 | 3632451O06Rik | NM_026142.1 | LA | 1.64 | 0.006 | LA | 1.63 | 0.015 | LA | 1.43 |  |
| 6590370 | 4631416L12Rik | NM_001081295.1 | RA | 0.58 | 0.001 | na | na | na | na | na | na |
| 4920162 | 4732429D16Rik | NM_145437.2 | RA | 0.56 | 0.001 | RA | 0.80 |  | RA | 0.89 |  |
| 2000768 | 4833412C05Rik |  | RA | 0.43 | 0.002 | na | na | na | na | na | na |
| 5050088 | 4930455G09Rik |  | RA | 0.52 | 0.001 | na | na | na | na | na | na |
| 4010278 | 4930544G21Rik | NM_172430.2 | RA | 0.89 |  | RA | 0.62 | 0.003 | RA | 0.87 |  |
| 6100561 | 4930555G01Rik | NM_175393.2 | RA | 0.59 |  | LA | 1.21 |  | RA | 0.63 | 0.002 |
| 7610348 | 4933425L21Rik |  | LA | 1.58 | 0.001 | na | na | na | na | na | na |
| 5570097 | 5330431K02Rik | NM_026606.1 | RA | 0.65 | 0.027 | na | na | na | na | na | na |
| 4640731 | 5330432B20Rik | XM_976590.1 | RA | 0.56 | 0.004 | na | na | na | na | na | na |
| 6770075 | 5430417L22Rik |  | RA | 0.61 | 0.000 | na | na | na | na | na | na |
| 650392 | 5430433G21Rik | XM_001480745.1 | RA | 0.61 | 0.005 | na | na | na | na | na | na |
| 2030367 | 5730410E15Rik | NM_178765.3 | LA | 1.51 |  | LA | 1.44 |  | LA | 1.55 | 0.004 |
| 2120309 | 6430537F04 |  | RA | 0.51 | 0.000 | na | na | na | na | na | na |
| 7160070 | 6430548M08Rik | | RA | 0.62 | 0.000 | na | na | na | na | na | na |
| 1190167 | 6430548M08Rik | | RA | 0.54 | 0.001 | RA | 0.87 |  | RA | 0.99 |  |
| 7510504 | 9130218E19Rik | XM_358331.1 | LA | 1.72 | 0.000 | na | na | na | na | na | na |
| 2630086 | 9630015D15Rik |  | RA | 0.76 |  | RA | 0.62 | 0.001 | RA | 0.81 |  |
| 1990500 | 9930105H17Rik |  | LA | 1.53 | 0.000 | na | na | na | na | na | na |
| 7380113 | A030007L17Rik | NM_026637.3 | LA | 1.59 | 0.001 | LA | 1.11 |  | LA | 1.25 |  |
| 3710209 | A2bp1 | NM_021477.2 | LA | 1.56 | 0.000 | LA | 1.01 |  | LA | 1.63 | 0.014 |
| 3800471 | A330102I10Rik |  | RA | 0.54 | 0.009 | na | na | na | na | na | na |
| 1070672 | A530088H08Rik | NM_178656.3 | RA | 0.62 | 0.004 | RA | 0.92 |  | LA | 1.12 |  |
| 6650121 | A930038C07Rik | NM_172399.2 | LA | 1.58 | 0.019 | LA | 1.14 |  | LA | 1.07 |  |
| 1740630 | AB182283 | NM_001081652.1 | LA | 1.53 | 0.005 | LA | 1.58 |  | LA | 1.30 |  |
| 6560594 | Abca8a | NM_153145.3 | RA | 0.66 | 0.021 | LA | 1.15 |  | RA | 0.93 |  |
| 7210136 | Abcb4 | NM_008830.1 | LA | 1.56 | 0.007 | LA | 1.88 | 0.002 | LA | 1.51 | 0.006 |
| 7550066 | Abra | NM_175456.4 | LA | 1.19 |  | LA | 1.65 | 0.007 | LA | 1.22 |  |
| 520367 | Accn2 | NM_009597.1 | RA | 0.58 | 0.000 | RA | 0.76 |  | RA | 0.63 | 0.004 |
| 5360674 | Ace | NM_207624.4 | LA | 1.67 | 0.003 | na | na | na | na | na | na |
| 7400328 | Acpl2 | NM_153420.2 | RA | 0.70 |  | RA | 0.57 | 0.001 | RA | 0.94 |  |
| 2030450 | Acta1 | NM_009606.2 | RA | 0.28 |  | LA | 11.53 | 0.020 | LA | 1.92 |  |
| 2140255 | Acta2 | NM_007392.2 | RA | 0.24 | 0.005 | LA | 1.22 |  | LA | 1.48 |  |
| 430068 | Acta2 | NM_007392.2 | RA | 0.26 | 0.004 | LA | 1.36 |  | LA | 1.32 |  |
| 6180554 | Actc1 | NM_009608.3 | LA | 1.02 |  | LA | 1.56 | 0.020 | RA | 0.88 |  |
| 4730458 | Adamts19 | NM_175506.3 | LA | 1.50 |  | RA | 0.86 |  | LA | 1.54 | 0.007 |
| 5080274 | Adamts8 | NM_013906.2 | LA | 2.05 | 0.002 | LA | 3.33 | 0.011 | LA | 1.21 |  |
| 4850521 | Adh1 | NM_007409.2 | RA | 0.68 |  | RA | 0.59 | 0.005 | RA | 0.89 |  |
| 110458 | Adh1 | NM_007409.2 | RA | 0.64 | 0.005 | RA | 0.47 | 0.001 | RA | 0.74 |  |
| 4540546 | Adh6b | XM_110439.5 | RA | 0.47 | 0.010 | na | na | na | na | na | na |
| 4280603 | Adm | NM_009627.1 | RA | 0.13 | 0.000 | RA | 0.13 | 0.000 | RA | 0.25 | 0.000 |
| 4250349 | Adprhl1 | NM_172750.3 | RA | 0.63 | 0.002 | RA | 0.73 |  | RA | 0.63 | 0.004 |
| 3120093 | Adprhl1 | NM_172750.1 | RA | 0.64 | 0.011 | RA | 0.68 |  | RA | 0.68 |  |
| 4480364 | Aebp1 | NM_009636.1 | RA | 0.63 | 0.007 | RA | 0.85 |  | RA | 0.76 |  |
| 2100035 | Ahsg | NM_013465.1 | RA | 0.46 | 0.003 | RA | 0.30 | 0.002 | RA | 0.58 | 0.008 |
| 6480326 | Ahsg | NM_013465.1 | RA | 0.49 | 0.003 | RA | 0.35 | 0.007 | RA | 0.50 | 0.001 |
| 4290605 | AI118078 | NM_172923.3 | LA | 1.51 | 0.000 | LA | 1.00 |  | LA | 1.20 |  |
| 1690600 | AI646023 | NM_198860.1 | RA | 0.77 |  | RA | 0.71 |  | RA | 0.66 | 0.002 |
| 3940561 | Aif1 | NM_019467.2 | RA | 0.63 | 0.001 | RA | 0.88 |  | RA | 0.85 |  |
| 4480541 | Alas2 | NM_009653.1 | LA | 1.02 |  | RA | 0.50 |  | RA | 0.51 | 0.006 |
| 2650154 | Aldh1b1 | NM_028270.4 | RA | 0.87 |  | RA | 0.59 | 0.045 | RA | 0.73 |  |
| 7560747 | Aldh1l2 | NM_153543.1 | LA | 1.64 | 0.004 | RA | 0.83 |  | LA | 1.15 |  |
| 5810367 | Aldh1l2 | NM_153543.1 | RA | 0.16 | 0.000 | RA | 0.38 | 0.001 | RA | 0.58 | 0.008 |
| 5050382 | Aldh3a1 | NM_007436.1 | LA | 1.82 | 0.001 | LA | 1.46 |  | LA | 1.24 |  |
| 5820286 | Alox5 | NM_009662.2 | LA | 1.75 | 0.000 | na | na | na | na | na | na |
| 6840471 | Alox8 | NM_009661.3 | RA | 0.56 | 0.002 | RA | 0.61 | 0.007 | RA | 0.99 |  |
| 4070328 | Amigo2 | NM_178114.3 | RA | 0.49 | 0.004 | na | na | na | na | na | na |
| 2470753 | Amigo2 | NM_178114.3 | RA | 0.50 | 0.006 | RA | 0.42 | 0.001 | RA | 0.50 | 0.004 |
| 4210189 | Angpt1 | NM_009640.3 | LA | 1.30 |  | LA | 1.28 |  | LA | 1.58 | 0.013 |
| 2630553 | Angptl1 | NM_028333.2 | LA | 1.72 | 0.014 | LA | 1.50 |  | LA | 1.18 |  |
| 1980605 | Angptl7 | NM_001039554.1 | RA | 0.61 | 0.009 | na | na | na | na | na | na |
| 6520451 | Angptl7 | NM_001039554.1 | RA | 0.32 | 0.001 | RA | 0.34 | 0.020 | RA | 0.53 | 0.006 |
| 2850064 | Ankrd23 | NM_153502.3 | RA | 0.57 | 0.009 | LA | 1.99 |  | RA | 0.82 |  |
| 2260692 | Antxr1 | NM_054041.2 | RA | 0.63 | 0.002 | RA | 0.69 |  | RA | 0.62 | 0.026 |
| 3190411 | Antxr1 | NM_054041.2 | RA | 0.65 | 0.005 | RA | 0.71 |  | RA | 0.59 | 0.001 |
| 3140400 | Aoc3 | NM_009675.1 | RA | 0.41 | 0.006 | RA | 0.80 |  | RA | 0.67 |  |
| 3190646 | Aox3 | NM_023617.2 | RA | 0.53 | 0.000 | RA | 0.54 | 0.006 | RA | 0.68 |  |
| 5820600 | Apoc1 | NM_007469.3 | RA | 0.36 | 0.000 | RA | 0.48 | 0.004 | RA | 0.66 |  |
| 1710066 | Apoc1 | NM_007469.3 | RA | 0.30 | 0.000 | RA | 0.30 | 0.004 | RA | 0.50 |  |
| 7210687 | Apoe | NM_009696.2 | RA | 0.66 | 0.039 | RA | 0.48 | 0.001 | RA | 0.57 | 0.002 |
| 5130138 | Appl2 | NM_145220.2 | RA | 0.79 |  | RA | 0.65 |  | RA | 0.55 | 0.010 |
| 1230612 | Aqp1 | NM_007472.2 | LA | 1.42 |  | LA | 1.40 |  | LA | 1.74 | 0.001 |
| 1580167 | Aqp1 | NM_007472.1 | LA | 1.56 | 0.001 | LA | 1.40 |  | LA | 1.80 | 0.001 |
| 4210139 | Aqp1 | NM_007472.1 | LA | 1.47 |  | LA | 1.51 |  | LA | 1.69 | 0.003 |
| 3170594 | Aqp7 | NM_007473.3 | LA | 1.48 |  | LA | 1.27 |  | LA | 1.53 | 0.002 |
| 3060180 | Arc | NM_018790.2 | RA | 0.59 | 0.015 | RA | 0.59 | 0.010 | RA | 0.61 | 0.001 |
| 830450 | Arl4a | NM_007487.3 | LA | 1.95 | 0.000 | LA | 1.46 |  | LA | 1.96 | 0.002 |
| 7150541 | Arl4a | NM_007487.3 | LA | 1.74 | 0.001 | LA | 1.46 |  | LA | 1.58 | 0.016 |
| 2630370 | Arpp21 | NM_033264.1 | RA | 0.41 | 0.000 | RA | 0.57 | 0.001 | RA | 0.55 | 0.000 |
| 4210273 | Arpp21 | NM_028755.2 | RA | 0.25 | 0.000 | RA | 0.47 | 0.000 | RA | 0.41 | 0.000 |
| 6040408 | Art5 | NM_007491.1 | LA | 2.24 | 0.000 | LA | 1.32 |  | LA | 1.53 | 0.003 |
| 1470386 | Asah3l | NM_139306.1 | RA | 0.56 | 0.004 | RA | 0.37 | 0.001 | RA | 0.50 | 0.001 |
| 3850577 | Ascc1 | NM_026937.1 | RA | 0.74 |  | RA | 0.45 | 0.035 | RA | 0.76 |  |
| 1260164 | Asns | NM_012055.3 | RA | 0.59 | 0.045 | RA | 0.68 |  | RA | 0.78 |  |
| 3440445 | Atoh8 | NM_153778.3 | RA | 0.53 | 0.000 | RA | 0.71 |  | RA | 0.70 |  |
| 780356 | Atp2a1 | NM_007504.2 | LA | 2.16 | 0.008 | LA | 2.48 |  | LA | 1.41 |  |
| 2630367 | AU021034 | NM_177629.2 | LA | 1.60 | 0.002 | LA | 1.09 |  | RA | 0.99 |  |
| 1440630 | Axin2 | NM_015732.3 | RA | 0.82 |  | RA | 0.60 | 0.007 | RA | 0.94 |  |
| 1780088 | B230317C12Rik | NM_019833.2 | LA | 1.58 | 0.000 | LA | 1.47 |  | LA | 1.38 |  |
| 5820239 | BB146404 | NM_178908.3 | RA | 0.48 | 0.002 | RA | 0.57 | 0.001 | RA | 0.75 |  |
| 3610025 | BC004728 | NM_174992.2 | RA | 0.65 | 0.002 | RA | 0.67 |  | RA | 0.74 |  |
| 7380048 | BC017612 | NM_133214.2 | LA | 1.49 |  | LA | 1.43 |  | LA | 1.54 | 0.001 |
| 1240576 | BC022687 | NM_145450.3 | RA | 0.45 | 0.000 | RA | 0.38 | 0.001 | RA | 0.49 | 0.000 |
| 1110646 | BC022713 | NM_177761.2 | RA | 0.54 | 0.001 | na | na | na | na | na | na |
| 6480554 | BC023892 | XM_994662.1 | RA | 0.66 | 0.002 | na | na | na | na | na | na |
| 5670612 | BC034076 | NM_177649.3 | LA | 1.60 | 0.002 | LA | 2.65 | 0.005 | LA | 1.26 |  |
| 1510494 | BC055107 | NM_183187.3 | RA | 0.92 |  | RA | 0.61 | 0.040 | RA | 0.95 |  |
| 6110008 | Bcl11b | NM_021399.2 | RA | 0.89 |  | RA | 0.62 | 0.006 | RA | 0.93 |  |
| 7040202 | Bcl2a1d |  | RA | 0.66 | 0.003 | na | na | na | na | na | na |
| 5820168 | Bcl2l11 | NM_207680.2 | RA | 0.84 |  | RA | 0.60 | 0.006 | RA | 0.76 |  |
| 650110 | Bcl7a | NM_029850.2 | RA | 0.89 |  | RA | 0.65 | 0.017 | RA | 0.92 |  |
| 7150243 | Bdh1 | NM_175177.3 | LA | 1.19 |  | LA | 1.99 | 0.045 | LA | 1.84 | 0.024 |
| 1510543 | Bmp10 | NM_009756.1 | RA | 0.04 | 0.000 | RA | 0.24 | 0.001 | RA | 0.08 | 0.001 |
| 4780035 | Bmp10 |  | RA | 0.07 | 0.000 | na | na | na | na | na | na |
| 940121 | Bmp3 | NM_173404.2 | LA | 1.88 | 0.005 | LA | 1.15 |  | LA | 1.61 | 0.005 |
| 5820148 | C030009J22Rik |  | RA | 0.28 | 0.000 | na | na | na | na | na | na |
| 580332 | C1qb | NM_009777.2 | RA | 0.59 | 0.001 | RA | 0.74 |  | RA | 0.62 | 0.010 |
| 5390110 | C1qtnf9 | NM_183175.3 | LA | 1.65 | 0.002 | LA | 1.90 | 0.021 | LA | 1.43 |  |
| 4040154 | C230098O21Rik | | LA | 1.52 | 0.000 | na | na | na | na | na | na |
| 5860347 | C3 | NM_009778.1 | LA | 1.62 | 0.003 | LA | 1.10 |  | LA | 1.35 |  |
| 7510243 | C4b | NM_009780.1 | RA | 0.66 | 0.001 | RA | 0.89 |  | RA | 0.72 |  |
| 2490079 | C85627 | NM_001033794.1 | LA | 1.57 |  | LA | 1.66 | 0.001 | LA | 1.23 |  |
| 4050026 | Cacna1h | NM_021415.3 | RA | 0.55 |  | RA | 0.44 | 0.031 | RA | 0.69 |  |
| 2650010 | Cadps | NM_001042617.1 | RA | 0.77 |  | RA | 0.58 | 0.004 | RA | 0.96 |  |
| 5670477 | Car11 | NM_009800.2 | RA | 0.77 |  | RA | 0.57 | 0.008 | RA | 0.79 |  |
| 6520347 | Casq2 | NM_009814.1 | LA | 1.15 |  | LA | 2.14 | 0.001 | LA | 1.30 |  |
| 4610072 | Cav1 | NM_007616.3 | LA | 1.33 |  | LA | 1.53 | 0.001 | LA | 1.42 |  |
| 3400148 | Ccbp2 | NM_021609.3 | LA | 1.52 | 0.001 | LA | 1.73 | 0.013 | LA | 1.26 |  |
| 5310471 | Ccdc3 | XM_129987.2 | RA | 0.90 |  | LA | 1.22 |  | LA | 1.85 | 0.026 |
| 6060520 | Ccl11 | NM_011330.2 | LA | 1.80 | 0.005 | LA | 2.37 | 0.006 | LA | 3.18 | 0.003 |
| 830273 | Ccl11 | NM_011330.1 | LA | 1.24 |  | LA | 1.32 |  | LA | 1.81 | 0.005 |
| 1710612 | Ccl21b | NM_011124.4 | LA | 8.74 | 0.000 | LA | 4.22 | 0.001 | LA | 2.72 | 0.000 |
| 4230315 | Ccl21c | NM_023052.1 | LA | 5.16 | 0.000 | LA | 3.08 | 0.007 | LA | 3.04 | 0.000 |
| 6370475 | Ccl21c |  | LA | 6.36 | 0.000 | na | na | na | na | na | na |
| 6940184 | Ccl4 | NM_013652.2 | RA | 0.65 | 0.008 | RA | 0.88 |  | RA | 0.85 |  |
| 1690768 | Ccl5 | NM_013653.2 | RA | 0.76 |  | RA | 0.65 | 0.028 | RA | 0.86 |  |
| 1580088 | Ccnd2 | NM_009829.3 | LA | 1.01 |  | LA | 1.80 | 0.031 | LA | 1.29 |  |
| 2120397 | Ccr5 | NM_009917.2 | RA | 0.64 | 0.001 | RA | 0.85 |  | RA | 0.85 |  |
| 1230161 | Cd163 | NM_053094.1 | RA | 0.66 | 0.001 | RA | 0.60 | 0.020 | RA | 0.63 | 0.010 |
| 7560255 | Cd207 | NM_144943.2 | RA | 0.12 | 0.000 | RA | 0.09 | 0.000 | RA | 0.18 | 0.000 |
| 5690056 | Cd207 | NM_144943.2 | RA | 0.06 | 0.000 | RA | 0.05 | 0.000 | RA | 0.09 | 0.000 |
| 5090309 | Cd209b | NM_026972.2 | LA | 1.42 |  | LA | 1.93 | 0.016 | LA | 1.27 |  |
| 2470307 | Cd209b | NM_026972.2 | LA | 1.19 |  | LA | 1.55 | 0.004 | LA | 1.23 |  |
| 6250497 | Cd209d | NM_130904.2 | LA | 1.39 |  | LA | 1.69 | 0.012 | LA | 1.13 |  |
| 6290093 | Cd209f | XM_284386.5 | LA | 1.95 | 0.006 | na | na | na | na | na | na |
| 4210427 | Cd248 | NM_054042.2 | LA | 1.42 |  | LA | 1.84 | 0.015 | LA | 1.55 | 0.011 |
| 6250040 | Cd300lg | NM_027987.2 | RA | 0.64 |  | RA | 0.62 | 0.038 | RA | 0.55 | 0.001 |
| 6290768 | Cd52 | NM_013706.1 | RA | 0.65 | 0.037 | RA | 0.85 |  | RA | 0.58 | 0.007 |
| 3360338 | Cd74 | NM_010545.3 | RA | 0.45 | 0.033 | RA | 0.70 |  | RA | 0.38 | 0.027 |
| 6770195 | Cd74 | NM_001042605.1 | RA | 0.47 | 0.033 | RA | 0.61 |  | RA | 0.34 | 0.023 |
| 7150377 | Cd83 | NM_009856.1 | LA | 1.57 | 0.000 | LA | 1.12 |  | LA | 1.36 |  |
| 4050075 | Cdh22 | NM_174988.2 | LA | 1.78 | 0.000 | LA | 1.47 |  | LA | 1.06 |  |
| 1410296 | Cdo1 | NM_033037.3 | RA | 0.63 |  | RA | 0.59 | 0.020 | RA | 0.79 |  |
| 2260639 | Cdv3 | NM_175833.1 | RA | 0.78 |  | RA | 0.54 | 0.001 | RA | 0.65 | 0.002 |
| 3360452 | Cdv3 | NM_175565.3 | RA | 0.71 |  | RA | 0.57 | 0.001 | RA | 0.75 |  |
| 4200195 | Cfi | NM_007686.2 | RA | 0.61 | 0.000 | RA | 0.70 |  | RA | 0.78 |  |
| 1770717 | Cfp | NM_008823.3 | RA | 0.56 | 0.000 | RA | 0.79 |  | RA | 0.49 | 0.001 |
| 3400431 | Chst4 |  | LA | 1.60 | 0.002 | na | na | na | na | na | na |
| 3780021 | Cidea | NM_007702.1 | RA | 0.49 | 0.002 | RA | 0.53 | 0.034 | RA | 0.85 |  |
| 1500427 | Cilp2 | NM_026818.1 | LA | 1.72 | 0.001 | LA | 1.21 |  | LA | 1.23 |  |
| 4610674 | Ckmt1 | NM_009897.2 | RA | 0.63 | 0.005 | RA | 0.55 | 0.013 | RA | 0.63 | 0.033 |
| 2600630 | Ckmt2 | NM_198415.2 | LA | 1.54 | 0.007 | LA | 1.40 |  | LA | 2.28 | 0.001 |
| 430427 | Ckmt2 | NM_198415.1 | LA | 1.55 | 0.001 | LA | 1.54 | 0.020 | LA | 2.30 | 0.000 |
| 270575 | Cldn10 | NM_023878.2 | LA | 1.58 | 0.001 | LA | 1.14 |  | LA | 1.20 |  |
| 2340056 | Cldn10 | NM_021386.3 | LA | 1.80 | 0.005 | LA | 1.20 |  | LA | 1.38 |  |
| 2350398 | Cldn23 | NM_027998.3 | RA | 0.40 |  | RA | 0.56 | 0.021 | RA | 0.53 |  |
| 3310026 | Clec3b | NM_011606.1 | LA | 1.78 | 0.003 | LA | 1.53 |  | LA | 1.52 | 0.015 |
| 870112 | Clec4b1 | NM_027218.1 | RA | 0.66 | 0.002 | LA | 1.08 |  | RA | 0.86 |  |
| 10170 | Clec4d | NM_010819.3 | RA | 0.60 | 0.002 | RA | 0.65 | 0.039 | RA | 0.73 |  |
| 1450333 | Clec7a | NM_020008.1 | RA | 0.53 | 0.000 | RA | 0.81 |  | RA | 0.78 |  |
| 6940753 | Cml3 | NM_053097.1 | RA | 0.65 | 0.025 | RA | 0.43 |  | RA | 0.84 |  |
| 6370148 | Cntn2 | NM_177129.5 | RA | 0.23 | 0.000 | na | na | na | na | na | na |
| 3850438 | Col15a1 | NM_009928.3 | LA | 1.26 |  | LA | 2.37 | 0.015 | LA | 1.50 |  |
| 4060102 | Col4a1 | NM_009931.1 | LA | 1.00 |  | LA | 2.23 | 0.035 | LA | 1.15 |  |
| 2260551 | Col4a2 | NM_009932.2 | LA | 1.01 |  | LA | 2.38 | 0.031 | LA | 1.14 |  |
| 7210041 | Col6a1 | NM_009933.2 | LA | 1.19 |  | LA | 1.88 | 0.016 | LA | 1.26 |  |
| 3170280 | Col6a1 | NM_009933.2 | LA | 1.12 |  | LA | 1.95 | 0.005 | LA | 1.22 |  |
| 1440189 | Col6a2 | NM_146007.1 | LA | 1.20 |  | LA | 1.97 | 0.010 | LA | 1.27 |  |
| 5490477 | Col8a1 | NM_007739.2 | LA | 1.79 | 0.005 | na | na | na | na | na | na |
| 4210376 | Col8a1 |  | LA | 1.74 | 0.001 | na | na | na | na | na | na |
| 4150196 | Cox10 | NM_178379.2 | LA | 1.09 |  | LA | 1.50 | 0.007 | LA | 1.24 |  |
| 1410739 | Cpa3 | NM_007753.1 | RA | 0.64 | 0.000 | RA | 0.76 |  | RA | 0.71 |  |
| 7650487 | Cpa3 | NM_007753.1 | RA | 0.65 | 0.004 | RA | 0.73 |  | RA | 0.64 | 0.005 |
| 3780102 | Crispld2 | NM_030209.2 | LA | 1.81 | 0.000 | LA | 1.50 | 0.039 | LA | 1.42 |  |
| 3710411 | Csdc2 | NM_145473.2 | RA | 0.80 |  | RA | 0.49 | 0.034 | RA | 0.89 |  |
| 1660474 | Csnk1e | NM_013767.5 | RA | 0.84 |  | RA | 0.64 | 0.001 | RA | 0.91 |  |
| 7150278 | Csrp2 | NM_007792.3 | RA | 0.44 | 0.000 | RA | 0.68 |  | RA | 0.63 | 0.033 |
| 7050324 | Cst12 | XM_918155.3 | RA | 0.49 | 0.003 | na | na | na | na | na | na |
| 5890347 | Cst9 | NM_009979.1 | RA | 0.40 | 0.000 | RA | 0.44 |  | RA | 0.14 | 0.001 |
| 6980273 | Ctdspl | NM_133710.1 | RA | 0.78 |  | RA | 0.64 | 0.002 | RA | 0.77 |  |
| 4010082 | Ctgf | NM_010217.1 | LA | 1.32 |  | LA | 2.81 | 0.008 | LA | 1.26 |  |
| 6130300 | Ctnnbl1 | NM_025680.3 | RA | 0.55 | 0.000 | RA | 0.47 | 0.003 | RA | 0.80 |  |
| 610414 | Ctsa | NM_001038492.1 | LA | 1.38 |  | LA | 1.75 | 0.005 | LA | 1.34 |  |
| 4200646 | Ctss | NM_021281.1 | RA | 0.63 | 0.014 | RA | 0.96 |  | RA | 0.80 |  |
| 6620376 | Cx3cr1 | NM_009987.2 | RA | 0.44 | 0.000 | RA | 0.99 |  | RA | 0.82 |  |
| 7550112 | Cxcl12 | NM_013655.2 | LA | 1.34 |  | LA | 2.57 | 0.007 | LA | 2.15 | 0.004 |
| 20088 | Cxcl12 | NM_001012477.1 | LA | 1.39 |  | LA | 1.31 |  | LA | 1.67 | 0.001 |
| 3060040 | Cxcl13 | NM_018866.1 | RA | 0.07 | 0.000 | RA | 0.07 | 0.000 | RA | 0.13 | 0.000 |
| 150746 | Cxcl14 | NM_019568.2 | LA | 3.75 | 0.000 | LA | 2.37 | 0.007 | LA | 2.54 | 0.005 |
| 3520349 | Cxcl14 | NM_019568.1 | LA | 2.29 | 0.000 | LA | 1.62 | 0.021 | LA | 1.49 |  |
| 4040524 | Cxcl4 | NM_019932.2 | RA | 0.76 |  | RA | 0.66 |  | RA | 0.61 | 0.002 |
| 4570397 | Cyb561 | NM_007805.4 | LA | 1.58 | 0.003 | LA | 1.58 | 0.014 | LA | 1.44 |  |
| 4250246 | Cyp1b1 | NM_009994.1 | RA | 0.62 | 0.005 | RA | 0.54 | 0.014 | RA | 0.65 | 0.001 |
| 6280133 | Cyp2e1 | NM_021282.2 | RA | 0.40 |  | RA | 0.19 | 0.004 | RA | 0.53 |  |
| 610491 | D12Ertd553e | NM_029758.3 | RA | 0.86 |  | RA | 0.66 | 0.004 | RA | 0.99 |  |
| 6980189 | D19Bwg1357e | NM_177474.4 | RA | 0.74 |  | RA | 0.63 | 0.039 | LA | 1.16 |  |
| 3140167 | D19Wsu162e | NM_146099.2 | RA | 0.87 |  | RA | 0.61 | 0.000 | RA | 0.85 |  |
| 5900743 | D430042O09Rik | NM_001081022.1 | RA | 0.63 | 0.012 | na | na | na | na | na | na |
| 7200377 | D630003M21Rik | NM_177657.3 | LA | 1.58 | 0.000 | LA | 1.18 |  | LA | 1.21 |  |
| 7320132 | D630003M21Rik | NM_177657.2 | LA | 2.70 | 0.000 | LA | 1.72 | 0.016 | LA | 1.85 | 0.001 |
| 510095 | D830041I17Rik |  | LA | 1.83 | 0.012 | na | na | na | na | na | na |
| 3310487 | Dapp1 | NM_011932.2 | LA | 1.59 | 0.003 | LA | 1.59 | 0.010 | LA | 1.06 |  |
| 2470719 | Dbh | NM_138942.2 | RA | 0.25 | 0.000 | RA | 0.19 | 0.000 | RA | 0.35 | 0.001 |
| 2060592 | Ddah1 |  | RA | 0.65 | 0.006 | RA | 0.68 |  | RA | 0.75 |  |
| 3120309 | Ddit4l | NM_030143.3 | LA | 6.50 | 0.000 | LA | 3.51 | 0.007 | LA | 3.89 | 0.000 |
| 3610139 | Ddr1 | NM_007584.2 | RA | 0.76 |  | RA | 0.65 | 0.007 | RA | 0.72 |  |
| 5090221 | Ddx26 |  | RA | 0.64 | 0.002 | na | na | na | na | na | na |
| 4060274 | Dlgap1 | NM_177639.5 | LA | 2.26 | 0.000 | na | na | na | na | na | na |
| 50521 | Dmkn | NM_172899.3 | RA | 0.85 |  | RA | 0.56 | 0.012 | RA | 0.72 |  |
| 3940682 | Dmkn | NM_172899.2 | RA | 0.82 |  | RA | 0.56 | 0.007 | RA | 0.68 |  |
| 1740364 | Dmn | NM_183312.3 | RA | 0.56 | 0.008 | LA | 1.18 |  | LA | 1.10 |  |
| 1410215 | Dmn | NM_183312.3 | RA | 0.63 | 0.002 | RA | 0.96 |  | RA | 0.85 |  |
| 6840356 | Dnajb6 | NM_001037940.1 | RA | 0.84 |  | RA | 0.90 |  | RA | 0.67 | 0.003 |
| 4920392 | Dok4 | NM_053246.2 | RA | 0.51 | 0.000 | RA | 0.60 | 0.003 | RA | 0.62 | 0.001 |
| 2350747 | Dpep1 | NM_007876.1 | RA | 0.58 | 0.020 | RA | 0.78 |  | RA | 0.96 |  |
| 4540379 | Dpysl3 | NM_009468.3 | RA | 0.65 | 0.001 | LA | 1.06 |  | LA | 1.08 |  |
| 4850301 | Drd2 | NM_010077.1 | LA | 1.74 | 0.002 | LA | 1.44 |  | LA | 1.08 |  |
| 460075 | Dusp26 | NM_025869.3 | RA | 0.56 | 0.011 | RA | 0.73 |  | LA | 1.05 |  |
| 7610398 | Dysf | NM_021469.2 | LA | 1.12 |  | LA | 1.89 | 0.001 | LA | 1.11 |  |
| 3780224 | E430002D04Rik | NM_172909.1 | LA | 1.01 |  | LA | 1.68 | 0.018 | LA | 1.15 |  |
| 1980095 | Ear11 | NM_053113.2 | LA | 1.76 | 0.023 | LA | 1.30 |  | LA | 1.15 |  |
| 5700722 | Ece1 | NM_199307.1 | RA | 0.90 |  | RA | 0.62 | 0.014 | RA | 0.73 |  |
| 1710242 | Ecm1 | NM_007899.1 | RA | 0.56 | 0.001 | RA | 0.54 | 0.002 | RA | 0.66 | 0.003 |
| 4210196 | Ednrb | NM_007904.3 | RA | 0.65 | 0.007 | RA | 0.99 |  | RA | 0.89 |  |
| 1400152 | Efnb1 | NM_010110.2 | RA | 0.75 |  | RA | 0.59 | 0.004 | RA | 0.73 |  |
| 2750326 | Egln3 | NM_028133.1 | RA | 0.48 | 0.000 | RA | 0.63 |  | RA | 0.66 | 0.003 |
| 20612 | Egr3 |  | LA | 1.79 | 0.007 | na | na | na | na | na | na |
| 5270021 | Emcn | NM_016885.1 | RA | 0.87 |  | RA | 0.65 | 0.013 | RA | 0.87 |  |
| 5910164 | Emilin2 | NM_145158.2 | RA | 0.35 | 0.000 | RA | 0.36 | 0.001 | RA | 0.45 | 0.002 |
| 5490026 | Emilin2 |  | RA | 0.37 | 0.000 | na | na | na | na | na | na |
| 1030411 | Emr1 | NM_010130.3 | RA | 0.65 | 0.001 | RA | 0.89 |  | RA | 0.74 |  |
| 5910239 | Eng | NM_007932.1 | RA | 0.67 |  | RA | 0.59 | 0.010 | RA | 0.63 | 0.005 |
| 1090767 | Eno2 | NM_013509.2 | RA | 0.61 | 0.000 | RA | 0.67 |  | RA | 0.73 |  |
| 3360162 | Enpp2 | NM_015744.1 | RA | 0.43 | 0.000 | RA | 0.42 | 0.002 | RA | 0.80 |  |
| 10446 | Enpp2 |  | RA | 0.50 | 0.004 | na | na | na | na | na | na |
| 160411 | Entpd2 | NM_009849.1 | LA | 1.87 | 0.001 | LA | 1.80 | 0.049 | LA | 2.23 | 0.001 |
| 7210040 | Epb4.1 | NM_183428.2 | RA | 0.90 |  | RA | 0.65 | 0.021 | RA | 0.79 |  |
| 6250154 | Epha7 | NM_010141.2 | LA | 1.57 | 0.001 | LA | 1.12 |  | LA | 1.20 |  |
| 7610301 | Epyc | NM_007884.2 | RA | 0.61 | 0.001 | RA | 0.81 |  | LA | 1.03 |  |
| 3420451 | Esm1 | NM_023612.3 | LA | 1.73 | 0.001 | LA | 1.44 |  | LA | 1.06 |  |
| 6040343 | Esr2 | NM_207707.1 | RA | 0.61 | 0.032 | RA | 0.80 |  | RA | 0.77 |  |
| 610528 | Extl3 | NM_018788.2 | RA | 0.72 |  | RA | 0.65 | 0.002 | RA | 0.77 |  |
| 6960451 | F13a1 | NM_028784.2 | LA | 1.71 | 0.017 | LA | 2.59 | 0.005 | LA | 2.08 | 0.001 |
| 4260195 | Fblim1 | NM_133754.3 | LA | 1.54 | 0.030 | LA | 1.89 | 0.004 | LA | 1.62 | 0.002 |
| 2690070 | Fbln1 | NM_010180.1 | RA | 0.88 |  | RA | 0.59 | 0.029 | LA | 1.01 |  |
| 1190041 | Fbln1 | NM_010180.1 | RA | 0.89 |  | RA | 0.53 | 0.022 | RA | 0.98 |  |
| 4230228 | Fbln2 | NM_001081437.1 | LA | 1.25 |  | LA | 2.08 | 0.001 | LA | 1.62 | 0.023 |
| 6040521 | Fbln2 | NM_001081437.1 | LA | 1.15 |  | LA | 1.77 | 0.003 | LA | 1.12 |  |
| 1110326 | Fcgr2b | NM_010187.2 | LA | 1.59 | 0.000 | LA | 1.44 |  | LA | 1.17 |  |
| 830632 | Fcgr4 | NM_144559.1 | RA | 0.59 | 0.000 | RA | 0.92 |  | RA | 0.79 |  |
| 7200341 | Fcna | NM_007995.3 | LA | 1.79 | 0.049 | LA | 2.64 | 0.005 | LA | 2.14 | 0.012 |
| 4540725 | Fetub | NM_021564.2 | RA | 0.81 |  | RA | 0.58 | 0.001 | RA | 0.78 |  |
| 130577 | Fgf10 | NM_008002.3 | RA | 0.42 | 0.000 | RA | 0.81 |  | RA | 0.79 |  |
| 2600286 | Fgf10 | NM_008002.3 | RA | 0.38 | 0.000 | RA | 0.79 |  | RA | 0.75 |  |
| 5390338 | Fgf12 | NM_010199.2 | LA | 1.04 |  | LA | 1.61 | 0.007 | LA | 1.05 |  |
| 160369 | Fgf12 | NM_010199.2 | LA | 1.04 |  | LA | 1.64 | 0.039 | RA | 0.89 |  |
| 4250437 | Fgfbp1 | NM_008009.2 | LA | 2.01 | 0.000 | LA | 1.19 |  | LA | 1.22 |  |
| 6060452 | Fgfr2 | NM_201601.2 | RA | 0.54 | 0.004 | na | na | na | na | na | na |
| 10639 | Fhl1 | NM_001077362.1 | RA | 0.85 |  | LA | 2.13 | 0.037 | LA | 1.04 |  |
| 5550309 | Fhl1 | NM_001077361.1 | RA | 0.86 |  | LA | 2.13 | 0.036 | LA | 1.08 |  |
| 5860521 | Figf | NM_010216.1 | LA | 1.05 |  | LA | 1.75 | 0.043 | LA | 1.09 |  |
| 4640446 | Fmo1 | NM_010231.2 | RA | 0.56 | 0.000 | RA | 0.69 |  | RA | 0.69 |  |
| 7650048 | Fpr2 | NM_008039.2 | RA | 0.65 | 0.000 | RA | 0.67 |  | RA | 0.68 |  |
| 6330181 | Frzb | NM_011356.4 | RA | 0.59 | 0.000 | RA | 0.81 |  | RA | 0.77 |  |
| 2120167 | Ftcd | NM_080845.1 | RA | 0.57 | 0.000 | RA | 0.66 | 0.006 | RA | 0.81 |  |
| 2470408 | Fxyd3 | NM_008557.1 | RA | 0.20 | 0.000 | RA | 0.36 | 0.000 | RA | 0.32 | 0.001 |
| 5570309 | Fzd1 | NM_021457.2 | RA | 0.64 | 0.011 | RA | 0.91 |  | LA | 1.00 |  |
| 6650333 | Galntl2 | NM_030166.1 | RA | 0.61 | 0.007 | na | na | na | na | na | na |
| 4760204 | Galntl2 | NM_030166.1 | RA | 0.51 | 0.000 | na | na | na | na | na | na |
| 2940681 | Gata2 | NM_008090.4 | RA | 0.91 |  | RA | 0.58 | 0.014 | RA | 0.81 |  |
| 6350678 | Gata6 | NM_010258.3 | RA | 0.90 |  | RA | 0.66 | 0.014 | RA | 0.79 |  |
| 1340711 | Gchfr | NM_177157.4 | LA | 1.67 | 0.005 | LA | 1.36 |  | LA | 1.26 |  |
| 770672 | Gja4 | NM_008120.2 | LA | 1.54 | 0.004 | LA | 1.61 | 0.017 | LA | 1.19 |  |
| 360133 | Gja5 | NM_008121.2 | RA | 0.98 |  | RA | 0.59 | 0.014 | LA | 1.18 |  |
| 4290204 | Gja5 | NM_008121.2 | LA | 1.13 |  | RA | 0.63 | 0.017 | LA | 1.01 |  |
| 4830382 | Gm1631 | NM_201366.1 | LA | 2.16 | 0.012 | LA | 1.80 | 0.000 | LA | 1.54 | 0.004 |
| 610672 | Gm1631 |  | LA | 2.86 | 0.006 | na | na | na | na | na | na |
| 2260327 | Gnao1 | NM_010308.3 | RA | 0.69 |  | RA | 0.65 | 0.017 | RA | 0.79 |  |
| 4880369 | Gnao1 | NM_010308.3 | RA | 0.67 |  | RA | 0.60 | 0.007 | RA | 0.70 |  |
| 2650743 | Gng13 | NM_022422.4 | RA | 0.29 | 0.000 | RA | 0.32 | 0.002 | RA | 0.38 | 0.007 |
| 2000288 | Gng8 | NM_010320.3 | RA | 0.60 | 0.000 | RA | 0.70 |  | RA | 0.74 |  |
| 7210039 | Gpc1 | NM_016696.3 | RA | 0.79 |  | RA | 0.62 | 0.004 | RA | 0.77 |  |
| 5900544 | Gpihbp1 | NM_026730.1 | RA | 0.95 |  | LA | 1.51 | 0.008 | RA | 0.98 |  |
| 4670228 | Gpnmb | NM_053110.3 | RA | 0.99 |  | LA | 1.57 | 0.039 | LA | 1.41 |  |
| 450743 | Gpr153 | NM_178406.2 | LA | 1.39 |  | LA | 1.55 | 0.004 | LA | 1.24 |  |
| 520010 | Gpr158 | NM_001004761.1 | LA | 1.54 | 0.010 | na | na | na | na | na | na |
| 6620044 | Gsg1 | NM_001080553.1 | RA | 0.55 | 0.006 | RA | 0.34 | 0.001 | RA | 0.61 |  |
| 6860377 | Gsg1 | NM_001080552.1 | RA | 0.62 | 0.010 | RA | 0.52 | 0.002 | RA | 0.79 |  |
| 1770598 | Gsg1l | XM_914623.2 | RA | 0.53 | 0.001 | na | na | na | na | na | na |
| 5700164 | Gsg1l | XM_914623.2 | RA | 0.21 | 0.004 | na | na | na | na | na | na |
| 4860646 | Gucy1a3 | NM_021896.4 | LA | 2.29 | 0.000 | LA | 2.36 | 0.001 | LA | 1.86 | 0.002 |
| 70008 | Gucy1a3 | NM_021896.4 | LA | 1.93 | 0.002 | LA | 2.11 | 0.001 | LA | 1.77 | 0.001 |
| 3440463 | Gucy1a3 | NM_021896.4 | LA | 1.62 | 0.001 | LA | 1.65 | 0.007 | LA | 1.39 |  |
| 650707 | H2-Aa | NM_010378.2 | RA | 0.51 |  | RA | 0.70 |  | RA | 0.50 | 0.034 |
| 5860435 | H2-Ab1 | NM_207105.1 | RA | 0.57 |  | RA | 0.87 |  | RA | 0.57 | 0.047 |
| 520072 | H2-Eb1 | NM_010382.2 | RA | 0.65 |  | LA | 1.12 |  | RA | 0.46 | 0.036 |
| 6560747 | H2-M2 | NM_008204.2 | RA | 0.64 | 0.002 | RA | 0.84 |  | RA | 0.82 |  |
| 6280692 | Hamp | NM_032541.1 | RA | 0.09 | 0.000 | RA | 0.06 | 0.002 | RA | 0.14 | 0.000 |
| 7330482 | Hamp2 | NM_183257.1 | RA | 0.11 | 0.000 | RA | 0.08 | 0.001 | RA | 0.11 | 0.000 |
| 2000398 | Hba-a1 | NM_008218.2 | LA | 1.15 |  | RA | 0.82 |  | RA | 0.56 | 0.031 |
| 580402 | Hbb-b2 | NM_016956.2 | LA | 1.07 |  | RA | 0.85 |  | RA | 0.50 | 0.004 |
| 2970324 | Hdc | NM_008230.4 | RA | 0.54 | 0.031 | RA | 0.49 | 0.042 | RA | 0.51 | 0.006 |
| 6510487 | Hebp1 | NM_013546.2 | RA | 0.85 |  | RA | 0.64 | 0.004 | RA | 0.81 |  |
| 7570598 | Hey1 | NM_010423.2 | RA | 0.40 | 0.000 | RA | 0.39 | 0.001 | RA | 0.45 | 0.002 |
| 4490445 | Hs3st1 | NM_010474.1 | LA | 1.22 |  | RA | 0.81 |  | LA | 1.50 | 0.010 |
| 4050369 | Hsd11b1 | NM_008288.2 | RA | 0.54 | 0.000 | RA | 0.68 |  | RA | 0.54 | 0.001 |
| 4570196 | Hsd11b1 | NM_008288.1 | RA | 0.54 | 0.000 | RA | 0.69 |  | RA | 0.56 | 0.003 |
| 2340301 | Hsd11b1 | NM_008288.1 | RA | 0.57 | 0.001 | RA | 0.75 |  | RA | 0.53 | 0.003 |
| 1710739 | Hsd3b2 | NM_153193.2 | RA | 0.86 |  | LA | 1.04 |  | RA | 0.59 | 0.038 |
| 6860739 | Hspa1l | NM_013558.1 | RA | 0.65 | 0.010 | RA | 0.75 |  | RA | 0.75 |  |
| 4040386 | Hspb6 | NM_001012401.1 | LA | 1.06 |  | LA | 1.65 | 0.001 | LA | 1.31 |  |
| 6980093 | Htra1 | NM_019564.1 | LA | 1.40 |  | LA | 1.62 | 0.001 | LA | 1.16 |  |
| 2970470 | Htra3 | NM_030127.2 | LA | 1.50 | 0.007 | LA | 1.30 |  | LA | 1.60 | 0.001 |
| 780546 | Htra3 | NM_030127.1 | LA | 2.06 | 0.000 | LA | 1.15 |  | LA | 2.19 | 0.002 |
| 6200692 | Id1 | NM_010495.2 | RA | 0.44 | 0.000 | RA | 0.55 | 0.009 | RA | 0.56 | 0.017 |
| 4490500 | Id2 | NM_010496.2 | RA | 0.53 | 0.018 | RA | 0.54 | 0.001 | RA | 0.78 |  |
| 1030519 | Id2 | NM_010496.2 | RA | 0.52 | 0.001 | RA | 0.35 | 0.003 | RA | 0.51 | 0.002 |
| 110471 | Id3 | NM_008321.1 | RA | 0.65 | 0.012 | RA | 0.54 | 0.005 | RA | 0.59 | 0.001 |
| 5050653 | Igf2 | NM_010514.2 | RA | 0.42 | 0.000 | RA | 0.50 | 0.024 | RA | 0.57 |  |
| 1820601 | Igfbp3 | NM_008343.2 | RA | 0.23 | 0.000 | na | na | na | na | na | na |
| 4920288 | Igfbp3 | NM_008343.2 | RA | 0.39 | 0.005 | RA | 0.47 | 0.002 | RA | 0.26 | 0.000 |
| 3400747 | Igfbp4 | NM_010517.3 | RA | 0.66 | 0.005 | RA | 0.73 |  | RA | 0.69 |  |
| 5080435 | Igfbp4 | NM_010517.2 | RA | 0.68 |  | RA | 0.65 | 0.005 | RA | 0.72 |  |
| 160100 | Igfbp5 | NM_010518.2 | RA | 0.63 | 0.003 | na | na | na | na | na | na |
| 5080292 | Igfbp5 | NM_010518.2 | RA | 0.58 | 0.002 | RA | 0.38 | 0.001 | RA | 0.69 |  |
| 4590414 | Igfbp5 |  | RA | 0.66 | 0.003 | na | na | na | na | na | na |
| 7380603 | Igfbp6 | NM_008344.2 | RA | 0.98 |  | RA | 0.55 | 0.009 | RA | 0.99 |  |
| 580767 | Igfbpl1 | NM_018741.1 | RA | 0.61 | 0.000 | RA | 0.83 |  | RA | 0.96 |  |
| 7510544 | Il13ra1 | NM_133990.4 | RA | 0.65 | 0.007 | RA | 0.82 |  | RA | 0.85 |  |
| 4640221 | Inmt | NM_009349.3 | LA | 2.63 | 0.001 | na | na | na | na | na | na |
| 2360050 | Inmt | NM_009349.1 | LA | 2.69 | 0.001 | LA | 1.83 |  | LA | 2.50 | 0.011 |
| 60703 | Irx3 |  | RA | 0.66 | 0.006 | RA | 0.91 |  | RA | 0.84 |  |
| 6960397 | Itga11 | NM_176922.4 | LA | 1.48 |  | LA | 1.67 |  | LA | 1.76 | 0.001 |
| 1780161 | Itga7 | NM_008398.2 | LA | 1.30 |  | LA | 1.86 | 0.001 | LA | 1.22 |  |
| 5360594 | Itgb1bp3 | NM_027120.2 | RA | 0.41 | 0.003 | na | na | na | na | na | na |
| 6290148 | Itgb1bp3 | NM_027120.2 | RA | 0.39 | 0.004 | na | na | na | na | na | na |
| 7610450 | Itpr2 | NM_019923.3 | RA | 0.94 |  | RA | 0.67 | 0.045 | RA | 0.91 |  |
| 1230068 | Kazald1 | NM_178929.3 | RA | 0.79 |  | RA | 0.60 | 0.002 | RA | 0.88 |  |
| 7570747 | Kbtbd10 | NM_001081087.1 | LA | 1.56 | 0.003 | na | na | na | na | na | na |
| 4010465 | Kcnc4 | NM_145922.2 | RA | 0.63 | 0.001 | RA | 0.84 |  | RA | 0.79 |  |
| 2190112 | Kcne1 | NM_008424.2 | RA | 0.61 | 0.013 | RA | 0.97 |  | RA | 0.89 |  |
| 6980196 | Kctd1 | NM_134112.4 | RA | 0.57 | 0.001 | na | na | na | na | na | na |
| 6400672 | Kif1b | NM_207682.2 | RA | 0.78 |  | RA | 0.65 | 0.006 | RA | 0.86 |  |
| 1510553 | Kif1b | NM_207682.2 | RA | 0.84 |  | RA | 0.63 | 0.020 | RA | 0.74 |  |
| 6200446 | Klk8 | NM_008940.2 | RA | 0.63 | 0.005 | RA | 0.62 | 0.005 | RA | 0.60 | 0.004 |
| 7330477 | Krt7 | NM_033073.2 | RA | 0.65 | 0.023 | RA | 0.61 | 0.007 | RA | 0.71 |  |
| 2940470 | Ky | NM_024291.3 | LA | 1.38 |  | LA | 1.28 |  | LA | 1.51 | 0.005 |
| 4850605 | Lamb3 | NM_008484.2 | LA | 1.76 | 0.000 | LA | 1.02 |  | LA | 1.87 | 0.002 |
| 5670440 | Lbh | NM_029999.3 | LA | 1.07 |  | LA | 1.89 | 0.037 | LA | 1.07 |  |
| 7040184 | Lgals7 | NM_008496.4 | LA | 1.58 | 0.043 | LA | 1.10 |  | LA | 1.01 |  |
| 1660575 | Lgi3 | NM_145219.2 | RA | 0.88 |  | RA | 0.56 | 0.027 | RA | 0.89 |  |
| 540730 | Lgr6 |  | LA | 1.51 | 0.008 | na | na | na | na | na | na |
| 7160022 | Lmcd1 | NM_144799.1 | RA | 0.91 |  | LA | 1.59 | 0.010 | LA | 1.44 |  |
| 2710347 | Lmcd1 | NM_144799.1 | RA | 0.89 |  | LA | 1.69 | 0.007 | LA | 1.36 |  |
| 6420376 | LOC100041504 | XM_001473258.1 | LA | 5.91 | 0.000 | LA | 2.88 | 0.003 | LA | 2.74 | 0.000 |
| 3140204 | LOC100046259 | XM_001476332.1 | RA | 0.17 | 0.000 | na | na | na | na | na | na |
| 6510162 | LOC100047200 | XM_001477632.1 | RA | 0.50 | 0.002 | RA | 0.72 |  | RA | 0.73 |  |
| 1400053 | LOC100047583 | XM_001479138.1 | RA | 0.62 | 0.012 | RA | 0.44 | 0.007 | RA | 0.88 |  |
| 4180437 | LOC100047619 | XR_033736.1 | RA | 0.67 |  | RA | 0.60 | 0.019 | RA | 0.85 |  |
| 2810280 | LOC100047856 | XM_001479297.1 | RA | 0.84 |  | RA | 0.94 |  | RA | 0.60 | 0.003 |
| 1660703 | LOC100048295 | XR_034465.1 | RA | 0.81 |  | RA | 0.49 | 0.002 | RA | 0.68 |  |
| 4760424 | LOC100048436 | XR_034485.1 | LA | 1.58 | 0.004 | LA | 1.67 | 0.010 | LA | 1.44 |  |
| 7160671 | LOC100048461 | XM_001480292.1 | RA | 0.64 | 0.003 | RA | 0.98 |  | RA | 0.93 |  |
| 2480296 | LOC100048554 | XM_001480890.1 | LA | 2.85 | 0.002 | LA | 2.96 | 0.005 | LA | 1.93 | 0.017 |
| 1990221 | LOC100048556 | XM_001480891.1 | LA | 1.84 | 0.002 | LA | 1.51 | 0.034 | LA | 1.34 |  |
| 2690754 | LOC211591 | XM_147649.1 | RA | 0.47 | 0.010 | na | na | na | na | na | na |
| 1740768 | LOC330517 | XM_286017.2 | LA | 1.55 | 0.000 | na | na | na | na | na | na |
| 4230193 | LOC381283 | XM_358544.1 | RA | 0.56 | 0.001 | na | na | na | na | na | na |
| 6180379 | LOC545013 | NM_001025085.1 | RA | 0.57 |  | LA | 1.22 |  | RA | 0.49 | 0.011 |
| 6130433 | LOC626152 | XR_032670.1 | RA | 0.78 |  | RA | 0.65 | 0.013 | RA | 0.89 |  |
| 780315 | LOC641240 | XM_918601.3 | RA | 0.54 |  | RA | 0.71 |  | RA | 0.45 | 0.034 |
| 4640717 | LOC669660 | XM_976375.1 | LA | 1.14 |  | LA | 2.76 | 0.024 | LA | 1.46 |  |
| 6060082 | LOC670044 | XM_978692.1 | RA | 0.38 | 0.000 | RA | 0.33 | 0.000 | RA | 0.37 | 0.001 |
| 6900328 | LOC676640 | XM_001003712.1 | RA | 0.54 | 0.000 | na | na | na | na | na | na |
| 5310162 | LOC677369 | XR_005046.1 | RA | 0.55 | 0.000 | na | na | na | na | na | na |
| 4830259 | Lrrc10 | NM_146242.2 | LA | 1.62 | 0.005 | LA | 1.54 | 0.007 | LA | 1.14 |  |
| 6900167 | Lrrc38 | XM_915579.2 | LA | 1.65 | 0.000 | na | na | na | na | na | na |
| 5390692 | Ltc4s | NM_008521.1 | LA | 1.44 |  | LA | 1.71 | 0.001 | LA | 1.37 |  |
| 4180475 | Lum | NM_008524.1 | LA | 1.20 |  | LA | 1.72 |  | LA | 1.54 | 0.012 |
| 1690187 | Ly6a | NM_010738.2 | LA | 1.24 |  | LA | 2.16 | 0.010 | LA | 1.50 | 0.007 |
| 5550671 | Ly6c1 | NM_010741.2 | LA | 1.26 |  | LA | 1.92 | 0.005 | LA | 1.61 | 0.005 |
| 6020358 | Lypd1 | NM_145100.3 | RA | 0.66 | 0.004 | RA | 0.44 | 0.001 | LA | 1.32 |  |
| 4880201 | Lyve1 | NM_053247.4 | RA | 0.78 |  | RA | 0.56 | 0.011 | RA | 0.65 | 0.009 |
| 5720609 | Lyz | NM_013590.2 | RA | 0.66 | 0.002 | RA | 0.81 |  | RA | 0.56 | 0.002 |
| 830240 | Lyz2 | NM_017372.3 | RA | 0.63 | 0.033 | RA | 0.71 |  | RA | 0.49 | 0.033 |
| 1940608 | Lyzs | NM_017372.2 | RA | 0.69 |  | RA | 0.88 |  | RA | 0.52 | 0.001 |
| 3710626 | Mapk10 | NM_009158.2 | LA | 2.75 | 0.000 | LA | 1.92 | 0.014 | LA | 1.50 | 0.003 |
| 6860543 | Mapre2 | NM_153058.3 | RA | 0.73 |  | RA | 0.63 | 0.005 | RA | 0.75 |  |
| 6040373 | Masp1 |  | LA | 3.14 | 0.000 | na | na | na | na | na | na |
| 3310491 | Mcam | NM_023061.1 | RA | 0.64 | 0.000 | LA | 1.01 |  | RA | 0.77 |  |
| 1980138 | Mdk | NM_010784.4 | RA | 0.71 |  | RA | 0.63 | 0.022 | RA | 0.77 |  |
| 2470465 | Mdk | NM_001012336.1 | RA | 0.67 |  | RA | 0.49 | 0.032 | RA | 0.61 | 0.003 |
| 5820369 | Mef2c | NM_025282.2 | LA | 1.10 |  | LA | 1.61 | 0.002 | LA | 1.21 |  |
| 4120300 | Megf10 | NM_001001979.1 | RA | 0.56 | 0.001 | na | na | na | na | na | na |
| 20075 | Mfap4 | NM_029568.2 | LA | 2.10 | 0.003 | LA | 2.47 | 0.004 | LA | 2.43 | 0.005 |
| 1740181 | Mfap5 | NM_015776.2 | LA | 1.21 |  | LA | 2.01 | 0.050 | LA | 1.47 |  |
| 1740546 | Mgl1 | NM_010796.2 | LA | 2.61 | 0.009 | LA | 3.98 | 0.001 | LA | 1.97 | 0.003 |
| 4150497 | Mgl2 | NM_145137.2 | LA | 1.81 | 0.012 | na | na | na | na | na | na |
| 5310328 | Mgll | NM_011844.3 | RA | 0.68 |  | RA | 0.79 |  | RA | 0.64 | 0.001 |
| 3890072 | Mgmt | NM_008598.2 | RA | 0.60 | 0.010 | RA | 0.53 | 0.000 | RA | 0.88 |  |
| 3990435 | Mgmt | NM_008598.1 | RA | 0.49 | 0.018 | na | na | na | na | na | na |
| 7400725 | Mgp | NM_008597.3 | LA | 1.32 |  | LA | 1.06 |  | LA | 1.58 | 0.010 |
| 6350564 | Mks1 | NM_001039684.2 | LA | 1.83 | 0.000 | na | na | na | na | na | na |
| 1300647 | Mlana | XM_129166.1 | RA | 0.79 |  | RA | 0.70 |  | RA | 0.48 | 0.004 |
| 7210440 | Mllt11 | NM_019914.3 | LA | 1.06 |  | LA | 1.62 | 0.044 | LA | 1.28 |  |
| 3870072 | Mmp2 | NM_008610.2 | LA | 1.45 |  | LA | 1.93 | 0.036 | LA | 1.68 | 0.001 |
| 1510750 | Mmp3 | NM_010809.1 | RA | 0.49 | 0.039 | RA | 0.99 |  | RA | 0.83 |  |
| 6180544 | Mmp3 | NM_010809.1 | RA | 0.63 | 0.037 | RA | 0.92 |  | RA | 0.78 |  |
| 1430747 | Mmrn1 | XM_284198.5 | LA | 2.24 | 0.000 | na | na | na | na | na | na |
| 4010221 | Mpeg1 | NM_010821.1 | RA | 0.59 | 0.026 | na | na | na | na | na | na |
| 610564 | Mrvi1 | NM_194464.2 | RA | 0.64 | 0.000 | RA | 0.59 | 0.001 | RA | 0.64 | 0.009 |
| 1230746 | Msc | NM_010827.2 | RA | 0.31 | 0.000 | RA | 0.40 | 0.003 | RA | 0.41 | 0.001 |
| 4210121 | Mthfd2 |  | LA | 1.64 | 0.005 | na | na | na | na | na | na |
| 1240372 | Mtr | XM_976165.1 | RA | 0.66 | 0.016 | na | na | na | na | na | na |
| 2120356 | Myl2 | NM_010861.3 | RA | 0.04 | 0.003 | RA | 0.47 |  | RA | 0.05 | 0.012 |
| 60202 | Myl3 | NM_010859.1 | RA | 0.84 |  | LA | 2.56 | 0.018 | RA | 0.90 |  |
| 50148 | Myl9 | XM_977718.2 | RA | 0.49 | 0.006 | na | na | na | na | na | na |
| 1170307 | Myl9 | XM_283793.2 | RA | 0.58 | 0.027 | na | na | na | na | na | na |
| 1240348 | Mylk | NM_139300.3 | RA | 0.87 |  | LA | 1.14 |  | LA | 1.93 | 0.021 |
| 430014 | Mylk | NM_139300.3 | RA | 0.86 |  | LA | 1.30 |  | LA | 1.86 | 0.020 |
| 7400463 | Myoc | NM_010865.2 | RA | 0.38 | 0.000 | RA | 0.54 |  | RA | 0.81 |  |
| 6760673 | Myocd | NM_145136.2 | RA | 0.99 |  | LA | 1.51 | 0.004 | LA | 1.23 |  |
| 4850731 | Nav1 | NM_173437.1 | LA | 1.17 |  | LA | 1.59 | 0.004 | LA | 1.34 |  |
| 4290544 | Nav1 | NM_173437.1 | LA | 1.13 |  | LA | 1.54 | 0.015 | LA | 1.29 |  |
| 2750114 | Ncapd3 | NM_178113.2 | RA | 0.64 | 0.013 | RA | 0.64 | 0.007 | RA | 0.80 |  |
| 4010292 | Nid1 | NM_010917.1 | LA | 1.29 |  | LA | 1.90 | 0.011 | LA | 1.42 |  |
| 6450138 | Nrn1l | NM_175024.3 | RA | 0.62 | 0.003 | RA | 0.62 | 0.003 | RA | 0.76 |  |
| 2710367 | Ntsr2 | NM_008747.2 | RA | 0.60 |  | RA | 0.51 | 0.005 | RA | 0.76 |  |
| 5310497 | Odz4 | NM_011858.3 | RA | 0.51 | 0.005 | na | na | na | na | na | na |
| 3060382 | Odz4 | NM_011858.3 | RA | 0.65 | 0.036 | RA | 0.90 |  | RA | 0.94 |  |
| 4590215 | Olfm1 | NM_019498.2 | LA | 1.64 | 0.003 | LA | 1.15 |  | LA | 1.82 | 0.001 |
| 6180129 | Olfm1 | NM_001038614.1 | LA | 1.84 | 0.000 | na | na | na | na | na | na |
| 6220386 | Olfm1 | NM_001038612.1 | LA | 1.78 | 0.001 | LA | 1.24 |  | LA | 1.65 | 0.002 |
| 1190202 | Olfml3 | NM_133859.2 | RA | 0.68 |  | RA | 0.57 | 0.004 | RA | 0.80 |  |
| 4150605 | Olig1 | NM_016968.4 | RA | 0.66 | 0.000 | RA | 0.77 |  | RA | 0.94 |  |
| 2940739 | Oxr1 | NM_130885.2 | LA | 1.67 | 0.001 | LA | 1.51 | 0.012 | LA | 1.48 |  |
| 4150424 | Pacrg | NM_027032.2 | RA | 0.81 |  | RA | 0.58 | 0.004 | RA | 0.91 |  |
| 380242 | Palm | NM_023128.2 | RA | 0.86 |  | RA | 0.65 | 0.020 | RA | 0.87 |  |
| 6380349 | Palm | NM_023128.2 | RA | 0.85 |  | RA | 0.63 | 0.017 | RA | 0.78 |  |
| 1230575 | Pcolce2 | NM_029620.2 | RA | 0.50 | 0.001 | RA | 0.64 | 0.008 | RA | 0.76 |  |
| 130224 | Pde1c | NM_001025568.1 | RA | 0.63 | 0.000 | na | na | na | na | na | na |
| 5690382 | Pdk3 | NM_145630.2 | RA | 0.80 |  | RA | 0.66 | 0.004 | RA | 0.81 |  |
| 650452 | Pdlim3 | NM_016798.3 | RA | 0.57 | 0.021 | RA | 0.72 |  | RA | 0.59 | 0.006 |
| 2000482 | Pdlim3 | NM_016798.2 | RA | 0.56 | 0.000 | RA | 0.72 |  | RA | 0.61 | 0.008 |
| 2940615 | Pdlim4 | NM_019417.2 | RA | 0.80 |  | RA | 0.51 | 0.001 | RA | 0.75 |  |
| 4070475 | Pdlim5 | NM_022554.1 | LA | 1.25 |  | LA | 2.22 | 0.004 | LA | 1.16 |  |
| 5860554 | Pdlim5 | NM_019809.1 | LA | 1.00 |  | LA | 2.73 | 0.012 | LA | 1.33 |  |
| 1030739 | Pecam1 | NM_008816.2 | LA | 1.06 |  | RA | 0.66 | 0.002 | RA | 0.88 |  |
| 840392 | Pftk1 | NM_011074.1 | RA | 0.66 | 0.001 | RA | 0.70 |  | RA | 0.74 |  |
| 4480180 | Phlda1 | NM_009344.1 | LA | 4.57 | 0.000 | LA | 3.82 | 0.010 | LA | 5.09 | 0.001 |
| 1230546 | Pi16 | NM_023734.3 | LA | 3.20 | 0.001 | LA | 3.19 | 0.014 | LA | 2.85 | 0.000 |
| 1990176 | Pilra |  | RA | 0.59 | 0.001 | na | na | na | na | na | na |
| 460364 | Pitpnc1 | NM_145823.2 | LA | 1.67 | 0.028 | LA | 1.41 |  | LA | 1.69 | 0.009 |
| 7320338 | Pitx2 | NM_011098.3 | LA | 4.74 | 0.000 | LA | 2.69 | 0.000 | LA | 3.32 | 0.000 |
| 1820746 | Pitx2 | NM_001042502.1 | LA | 15.43 | 0.000 | LA | 8.07 | 0.000 | LA | 10.27 | 0.000 |
| 6550600 | Pkig | NM_011106.2 | LA | 1.50 |  | LA | 1.89 | 0.024 | LA | 2.12 | 0.001 |
| 2900435 | Pla2g5 | NM_011110.3 | RA | 0.51 | 0.000 | RA | 0.61 | 0.049 | RA | 0.61 | 0.003 |
| 5860243 | Plac8 | NM_139198.1 | RA | 0.65 | 0.009 | LA | 1.04 |  | RA | 0.65 | 0.036 |
| 1240039 | Plcd3 | NM_152813.2 | RA | 0.85 |  | RA | 0.65 | 0.026 | RA | 0.69 |  |
| 4570224 | Popdc2 | NM_022318.2 | LA | 1.19 |  | LA | 2.05 | 0.009 | LA | 1.29 |  |
| 3890273 | Popdc2 | NM_022318.2 | LA | 1.22 |  | LA | 1.95 | 0.009 | LA | 1.00 |  |
| 4490309 | Ppap2a | NM_008247.2 | LA | 1.36 |  | LA | 1.64 | 0.020 | LA | 1.25 |  |
| 1300288 | Ppp1r1a | NM_021391.3 | LA | 2.03 | 0.001 | LA | 1.42 |  | LA | 1.08 |  |
| 7200524 | Ppp1r1b | NM_144828.1 | LA | 4.08 | 0.000 | LA | 3.65 | 0.006 | LA | 3.38 | 0.001 |
| 5490379 | Ppp1r1b | NM_144828.1 | LA | 1.66 | 0.002 | LA | 1.43 |  | LA | 1.28 |  |
| 7510129 | Prkar2b | NM_011158.3 | RA | 0.51 | 0.003 | na | na | na | na | na | na |
| 5560722 | Prlr | NM_011169.4 | LA | 1.78 | 0.001 | LA | 1.52 |  | LA | 1.67 | 0.001 |
| 3140706 | Prox1 | NM_008937.2 | LA | 1.51 | 0.000 | LA | 1.24 |  | LA | 1.33 |  |
| 2260010 | Psat1 | NM_177420.1 | LA | 1.82 | 0.000 | LA | 2.69 | 0.004 | LA | 1.57 | 0.001 |
| 5570672 | Ptgds | NM_008963.1 | RA | 0.22 | 0.000 | RA | 0.19 | 0.001 | RA | 0.33 | 0.001 |
| 3990026 | Ptpn11 | NM_011202.2 | RA | 0.73 |  | RA | 0.61 | 0.002 | RA | 0.88 |  |
| 4880431 | Ptpn11 | NM_011202.2 | RA | 0.69 |  | RA | 0.57 | 0.002 | RA | 0.86 |  |
| 4640626 | Ptpn11 | NM_011202.2 | RA | 0.75 |  | RA | 0.61 | 0.001 | RA | 0.80 |  |
| 5340064 | Pvalb | NM_013645.3 | RA | 0.59 | 0.007 | RA | 0.54 |  | RA | 0.69 |  |
| 5090187 | Pxmp2 | NM_008993.2 | LA | 1.55 | 0.002 | LA | 1.32 |  | LA | 1.34 |  |
| 1030142 | Pygl | NM_133198.1 | RA | 0.56 | 0.040 | RA | 0.85 |  | RA | 0.71 |  |
| 1780619 | Rab32 | NM_026405.3 | RA | 0.57 | 0.004 | RA | 0.62 |  | RA | 0.61 | 0.004 |
| 240543 | Rab33a | NM_011228.1 | RA | 0.47 | 0.000 | RA | 0.67 |  | RA | 0.80 |  |
| 4010243 | Ramp1 | NM_016894.2 | LA | 2.42 | 0.000 | LA | 1.82 | 0.010 | LA | 1.73 |  |
| 1690035 | Ramp2 | NM_019444.2 | RA | 0.87 |  | RA | 0.84 |  | RA | 0.66 | 0.004 |
| 6060367 | Rarres2 | NM_027852.1 | LA | 1.09 |  | RA | 0.58 | 0.012 | LA | 1.09 |  |
| 1660152 | Rasl10a | NM_145216.3 | RA | 0.67 |  | RA | 0.64 | 0.009 | RA | 0.89 |  |
| 2370373 | Rbm19 | NM_028762.1 | RA | 0.76 |  | RA | 0.60 | 0.001 | RA | 0.64 | 0.002 |
| 7400537 | Rbp7 | NM_022020.2 | LA | 1.32 |  | LA | 1.75 | 0.014 | LA | 1.44 |  |
| 7610484 | Reln | NM_011261.2 | LA | 2.39 | 0.000 | LA | 2.30 | 0.000 | LA | 2.55 | 0.001 |
| 6770050 | Rerg | NM_181988.1 | LA | 1.93 | 0.001 | LA | 1.14 |  | LA | 1.06 |  |
| 990100 | Rfx2 | NM_009056.1 | RA | 0.83 |  | RA | 0.66 | 0.003 | RA | 0.75 |  |
| 2760189 | Rgs17 | NM_019958.3 | RA | 0.66 | 0.011 | na | na | na | na | na | na |
| 1190139 | Rgs17 |  | RA | 0.65 | 0.006 | RA | 0.75 |  | RA | 0.90 |  |
| 4780128 | Rgs4 | NM_009062.3 | RA | 0.93 |  | LA | 1.55 | 0.011 | LA | 1.31 |  |
| 3170053 | Rgs5 | NM_009063.2 | LA | 1.34 |  | LA | 1.24 |  | LA | 2.14 | 0.006 |
| 2340059 | Rgs6 | NM_015812.3 | RA | 0.46 | 0.000 | RA | 0.77 |  | RA | 0.61 | 0.001 |
| 7000121 | Rgs7 | NM_011880.2 | LA | 1.54 | 0.000 | LA | 1.73 | 0.007 | LA | 1.18 |  |
| 1340220 | Rhbdl3 | NM_139228.2 | LA | 1.61 | 0.003 | LA | 1.45 |  | LA | 1.41 |  |
| 6130743 | Ror2 | NM_013846.3 | RA | 0.59 | 0.000 | RA | 0.74 |  | RA | 0.80 |  |
| 6040064 | Rprm | NM_023396.4 | LA | 3.12 | 0.000 | LA | 1.21 |  | LA | 1.46 |  |
| 1470731 | Ryr3 | XM_130497.2 | RA | 0.27 | 0.000 | na | na | na | na | na | na |
| 3360646 | Ryr3 | NM_177652.2 | RA | 0.35 | 0.000 | RA | 0.33 | 0.001 | RA | 0.37 | 0.001 |
| 160377 | Scara5 | NM_028903.1 | LA | 3.81 | 0.000 | LA | 4.57 | 0.002 | LA | 4.13 | 0.000 |
| 3890274 | Scd1 | NM_009127.3 | RA | 0.47 |  | RA | 0.65 | 0.011 | RA | 0.76 |  |
| 7000328 | Scgb1c1 | NM_001099742.1 | LA | 1.69 | 0.031 | na | na | na | na | na | na |
| 1190121 | scl000959.1_2 | BC059087.1 | RA | 0.60 | 0.006 | na | na | na | na | na | na |
| 6220543 | Scn4b | NM_001013390.2 | LA | 1.60 | 0.021 | LA | 2.28 |  | LA | 2.44 | 0.000 |
| 4290259 | Sds | NM_145565.1 | LA | 1.32 |  | RA | 0.58 |  | LA | 1.56 | 0.016 |
| 2340519 | Sept6 | NM_019942.4 | RA | 0.71 |  | RA | 0.65 | 0.003 | RA | 0.73 |  |
| 2680370 | Sept6 | NM_019942.2 | RA | 0.74 |  | RA | 0.57 | 0.001 | RA | 0.81 |  |
| 1050092 | Serpina3g | NM_009251.1 | RA | 0.72 |  | RA | 0.78 |  | RA | 0.60 | 0.003 |
| 380639 | Sertad4 | NM_198247.1 | RA | 0.98 |  | RA | 0.60 | 0.003 | RA | 0.87 |  |
| 6020725 | Setd8 | NM_030241.2 | LA | 1.21 |  | LA | 1.79 | 0.003 | LA | 1.34 |  |
| 1690114 | Setd8 | NM_030241.2 | LA | 1.18 |  | LA | 1.60 | 0.015 | LA | 1.25 |  |
| 4230446 | Sfrp2 | NM_009144.1 | RA | 0.36 | 0.003 | RA | 0.88 |  | RA | 0.78 |  |
| 1400689 | Sh3gl2 | NM_019535.2 | LA | 1.87 | 0.000 | LA | 1.71 | 0.025 | LA | 2.28 | 0.000 |
| 3360092 | Shox2 | NM_013665.1 | RA | 0.52 | 0.004 | RA | 0.95 |  | RA | 0.94 |  |
| 3800274 | Skil | NM_001039090.1 | RA | 0.77 |  | RA | 0.66 | 0.012 | RA | 0.82 |  |
| 7510291 | Slamf9 | NM_029612.3 | LA | 1.19 |  | LA | 1.75 | 0.049 | LA | 1.13 |  |
| 3190246 | Slc13a4 | NM_172892.1 | RA | 0.70 |  | RA | 0.41 | 0.004 | RA | 0.60 | 0.002 |
| 3290427 | Slc15a3 | NM_023044.1 | RA | 0.58 | 0.000 | RA | 0.77 |  | RA | 0.63 | 0.001 |
| 3120427 | Slc16a6 | NM_134038.2 | LA | 1.68 | 0.009 | LA | 1.31 |  | LA | 1.04 |  |
| 4250100 | Slc22a1 | NM_009202.4 | LA | 1.07 |  | RA | 0.54 | 0.036 | RA | 0.96 |  |
| 6840494 | Slc23a3 | NM_194333.3 | RA | 0.85 |  | RA | 0.60 | 0.026 | RA | 0.90 |  |
| 3130204 | Slc24a2 | NM_172426.2 | LA | 3.78 | 0.000 | na | na | na | na | na | na |
| 4280131 | Slc24a3 | NM_053195.2 | LA | 1.09 |  | LA | 1.21 |  | LA | 1.82 | 0.001 |
| 6350044 | Slc2a1 | NM_011400.2 | RA | 0.80 |  | RA | 0.58 | 0.014 | RA | 0.90 |  |
| 3710367 | Slc41a3 | NM_027868.2 | LA | 1.74 | 0.000 | LA | 2.01 | 0.004 | LA | 1.71 | 0.001 |
| 6550500 | Slc41a3 | NM_001037493.1 | LA | 1.71 | 0.000 | LA | 2.02 | 0.007 | LA | 1.86 | 0.001 |
| 7320349 | Slc7a7 | NM_011405.3 | RA | 0.86 |  | RA | 0.59 | 0.033 | RA | 0.72 |  |
| 7040100 | Slco2a1 | NM_033314.2 | RA | 0.82 |  | RA | 0.65 | 0.014 | RA | 0.78 |  |
| 3120546 | Slco2b1 | NM_175316.3 | LA | 2.86 | 0.000 | LA | 2.40 | 0.007 | LA | 2.17 | 0.004 |
| 430408 | Slit2 | NM_178804.2 | LA | 1.03 |  | LA | 2.13 | 0.010 | RA | 0.85 |  |
| 7550468 | Slurp1 | NM_020519.1 | RA | 0.53 | 0.000 | RA | 0.84 |  | RA | 0.84 |  |
| 1450672 | Smad6 | NM_008542.2 | RA | 0.72 |  | RA | 0.60 | 0.003 | RA | 0.59 | 0.010 |
| 1030044 | Smarcd3 | NM_025891.3 | RA | 0.52 | 0.000 | RA | 0.48 | 0.007 | RA | 0.48 | 0.001 |
| 4010632 | Snai2 | NM_011415.2 | RA | 0.58 | 0.001 | na | na | na | na | na | na |
| 4210129 | Socs2 | NM_007706.3 | LA | 1.49 |  | LA | 1.61 | 0.017 | LA | 1.23 |  |
| 4390037 | Sox9 |  | RA | 0.60 | 0.001 | na | na | na | na | na | na |
| 3370703 | Spsb4 | NM_145134.2 | LA | 1.37 |  | LA | 1.66 | 0.004 | LA | 1.47 |  |
| 3400414 | Srl | NM_175347.4 | RA | 0.90 |  | LA | 2.05 | 0.014 | LA | 1.30 |  |
| 6380452 | Srpx | NM_016911.4 | RA | 0.62 | 0.000 | RA | 0.69 |  | RA | 0.84 |  |
| 6350474 | Srpx | NM_016911.4 | RA | 0.52 | 0.000 | RA | 0.63 | 0.005 | RA | 0.69 |  |
| 2120195 | St5 | NM_029811.2 | RA | 0.62 | 0.000 | RA | 0.76 |  | RA | 0.80 |  |
| 2690300 | St8sia5 | NM_153124.1 | LA | 1.59 | 0.000 | na | na | na | na | na | na |
| 3120747 | Stc1 | NM_009285.3 | RA | 0.65 | 0.004 | na | na | na | na | na | na |
| 3120521 | Stk32b | NM_022416.1 | RA | 0.88 |  | RA | 0.66 | 0.004 | RA | 0.68 |  |
| 6770189 | Syn2 | NM_013681.1 | LA | 1.90 | 0.000 | LA | 1.84 | 0.006 | LA | 2.38 | 0.003 |
| 3710563 | Sytl2 | NM_001040088.1 | RA | 0.66 | 0.001 | RA | 0.66 | 0.006 | RA | 0.69 |  |
| 6660500 | Tbrg4 | NM_134011.1 | LA | 1.13 |  | LA | 1.52 | 0.014 | RA | 0.99 |  |
| 830392 | Tbx20 | NM_194263.1 | RA | 0.56 | 0.000 | RA | 0.96 |  | RA | 0.96 |  |
| 2370358 | Tbx20 | NM_194263.1 | RA | 0.57 | 0.002 | RA | 0.85 |  | RA | 0.76 |  |
| 1090598 | Tbx20 |  | RA | 0.59 | 0.007 | na | na | na | na | na | na |
| 2970687 | Tbx3 | NM_011535.2 | RA | 0.54 | 0.011 | RA | 0.82 |  | RA | 0.76 |  |
| 5490563 | Tcf21 | NM_011545.1 | RA | 0.54 | 0.001 | RA | 0.51 | 0.003 | RA | 0.51 | 0.005 |
| 1230767 | Thbd | NM_009378.2 | LA | 1.32 |  | LA | 1.13 |  | LA | 1.56 | 0.006 |
| 7040201 | Thy1 | NM_009382.3 | LA | 1.44 |  | LA | 1.63 | 0.017 | LA | 1.04 |  |
| 3840367 | Timd4 | NM_178759.4 | RA | 0.61 | 0.001 | RA | 0.67 |  | RA | 0.76 |  |
| 6110068 | Timm10 | NM_013899.1 | LA | 1.38 |  | LA | 1.84 | 0.017 | LA | 1.04 |  |
| 1400309 | Timp3 | NM_011595.2 | RA | 0.73 |  | RA | 0.57 | 0.005 | RA | 0.81 |  |
| 2470608 | Timp4 | NM_080639.3 | LA | 1.93 | 0.007 | LA | 2.77 | 0.001 | LA | 2.33 | 0.001 |
| 3710491 | Tm4sf5 | NM_029360.1 | LA | 1.62 | 0.001 | LA | 1.09 |  | LA | 1.23 |  |
| 5910408 | Tmc7 | NM_172476.4 | LA | 1.65 | 0.001 | LA | 1.22 |  | LA | 1.22 |  |
| 2230070 | Tmem108 | NM_178638.2 | RA | 0.32 | 0.000 | RA | 0.26 | 0.001 | RA | 0.41 | 0.001 |
| 3370021 | Tmem176a | NM_025326.2 | RA | 0.83 |  | RA | 0.67 | 0.005 | RA | 0.75 |  |
| 3450180 | Tmem176b | NM_023056.3 | RA | 0.76 |  | RA | 0.59 | 0.002 | RA | 0.74 |  |
| 3290471 | Tmem38a | NM_144534.1 | LA | 1.58 | 0.000 | LA | 1.59 | 0.027 | LA | 1.28 |  |
| 2750594 | Tmem51 | NM_145402.3 | RA | 0.77 |  | RA | 0.60 | 0.008 | RA | 0.71 |  |
| 2750184 | Tmem86a | NM_026436.3 | RA | 0.73 |  | RA | 0.47 | 0.007 | RA | 0.65 | 0.002 |
| 4250148 | Tmod4 | NM_016712.2 | LA | 1.67 | 0.009 | LA | 1.05 |  | LA | 1.21 |  |
| 4210446 | Tmod4 | NM_016712.2 | LA | 1.62 | 0.007 | LA | 1.01 |  | LA | 1.17 |  |
| 2030397 | Tmod4 | NM_016712.2 | LA | 1.52 | 0.022 | LA | 1.04 |  | LA | 1.13 |  |
| 1770541 | Tnfrsf12a | NM_013749.1 | RA | 0.64 | 0.008 | LA | 1.44 |  | LA | 1.04 |  |
| 2060546 | Tnfrsf19 | NM_013869.3 | RA | 0.75 |  | RA | 0.54 | 0.008 | RA | 0.87 |  |
| 2260066 | Tnni2 | NM_009405.2 | LA | 3.78 | 0.000 | LA | 2.60 | 0.019 | LA | 2.33 | 0.001 |
| 620064 | Tnnt1 | NM_011618.1 | LA | 1.52 | 0.023 | LA | 2.05 | 0.005 | LA | 1.51 |  |
| 110707 | Tnnt3 | NM_011620.2 | RA | 0.61 | 0.003 | RA | 0.98 |  | RA | 0.92 |  |
| 1070088 | Tpm2 | NM_009416.3 | LA | 1.11 |  | LA | 2.03 | 0.007 | LA | 1.41 |  |
| 7040044 | Trf | NM_133977.2 | RA | 0.66 |  | RA | 0.43 | 0.003 | RA | 0.70 |  |
| 5570367 | Tsen2 | NM_199033.1 | RA | 0.82 |  | RA | 0.60 | 0.002 | RA | 0.86 |  |
| 4880026 | Tspan17 | NM_028841.1 | LA | 1.24 |  | LA | 1.52 | 0.010 | LA | 1.22 |  |
| 3610170 | Ttll1 | NM_178869.3 | RA | 0.65 | 0.021 | RA | 0.71 |  | RA | 0.80 |  |
| 7100180 | Ttll1 | NM_178869.2 | RA | 0.61 | 0.008 | RA | 0.64 | 0.012 | RA | 0.91 |  |
| 1500301 | Tuba8 | NM_017379.1 | RA | 0.96 |  | LA | 1.51 | 0.020 | RA | 0.97 |  |
| 1030133 | Tubb2b | NM_023716.2 | LA | 2.10 | 0.001 | LA | 2.63 |  | LA | 2.31 | 0.001 |
| 6330538 | Ubxd3 | NM_178671.3 | RA | 0.64 | 0.004 | RA | 0.74 |  | RA | 0.89 |  |
| 2350131 | Ugt1a10 | NM_201641.2 | RA | 0.61 | 0.014 | na | na | na | na | na | na |
| 6330372 | Ugt1a10 | NM_201641.2 | RA | 0.60 | 0.016 | na | na | na | na | na | na |
| 1110048 | Unc13b | NM_001081413.1 | LA | 1.74 | 0.001 | LA | 1.47 |  | LA | 1.34 |  |
| 2030497 | Unc5b | NM_029770.2 | RA | 0.50 | 0.000 | RA | 0.77 |  | RA | 0.61 | 0.031 |
| 520333 | Upk3b | NM_175309.3 | RA | 0.78 |  | RA | 0.44 | 0.001 | RA | 0.82 |  |
| 4040243 | Upk3b | NM_175309.3 | RA | 0.76 |  | RA | 0.58 | 0.003 | RA | 0.74 |  |
| 6270670 | Upk3b | NM_175309.3 | RA | 0.83 |  | RA | 0.45 | 0.001 | RA | 0.66 |  |
| 2060725 | Uts2d | NM_198166.3 | LA | 2.65 | 0.000 | LA | 4.99 | 0.007 | LA | 2.30 | 0.026 |
| 1660368 | Vit | NM_028813.1 | RA | 0.48 | 0.000 | RA | 0.54 | 0.004 | RA | 0.72 |  |
| 6040356 | Vsig4 | NM_177789.4 | RA | 0.06 | 0.000 | RA | 0.17 | 0.000 | RA | 0.13 | 0.000 |
| 2760646 | Vsnl1 | NM_012038.3 | RA | 0.34 | 0.000 | RA | 0.48 | 0.008 | RA | 0.75 |  |
| 4040674 | Vstm2b | NM_021387.3 | LA | 1.20 |  | RA | 0.80 |  | LA | 1.52 | 0.001 |
| 6770671 | Vtn | NM_011707.1 | LA | 2.00 | 0.000 | LA | 2.14 | 0.001 | LA | 1.69 | 0.000 |
| 7510414 | Vwf | NM_011708.3 | RA | 0.56 | 0.000 | RA | 0.49 | 0.002 | RA | 0.59 | 0.001 |
| 1410279 | Wasf1 | NM_031877.2 | RA | 0.71 |  | RA | 0.64 | 0.003 | RA | 0.75 |  |
| 70601 | Wfdc2 | NM_026323.2 | LA | 1.81 | 0.019 | LA | 1.28 |  | LA | 1.14 |  |
| 4280477 | Wisp2 | NM_016873.1 | RA | 0.38 | 0.000 | RA | 0.84 |  | RA | 0.49 | 0.003 |
| 6100209 | Wnk4 | NM_175638.2 | LA | 1.91 | 0.000 | na | na | na | na | na | na |
| 4830008 | Wnt2 | NM_023653.4 | RA | 0.64 |  | RA | 0.53 | 0.001 | RA | 0.65 | 0.010 |
| 2070376 | Xpnpep2 | NM_133213.2 | LA | 3.21 | 0.000 | LA | 1.65 | 0.047 | LA | 1.47 |  |
| 6760474 | Zcchc5 | NM_199468.1 | RA | 0.52 | 0.000 | RA | 0.82 |  | RA | 0.79 |  |
| 3450427 | Zdhhc9 | NM_172465.3 | LA | 1.44 |  | LA | 1.83 | 0.006 | LA | 1.24 |  |
| 3140349 | Zfp36l1 | NM_007564.4 | RA | 0.89 |  | RA | 0.65 | 0.001 | RA | 0.84 |  |
| 1240682 | Zfp521 | NM_181326.3 | RA | 0.70 |  | RA | 0.54 | 0.002 | RA | 0.74 |  |
