# Supplementary material for: Systematic Analysis of Gene Expression Differences between Left and Right Atria in Different Mouse Strains and in Human Atrial Tissue
Source: PLoS One. 2011 Oct 19;6(10):e26389. doi: 10.1371/journal.pone.0026389 (PMC3198471; doi:10.1371/journal.pone.0026389)
Supplement: Table S2 — Gene set enrichment analysis results. (DOCX) [file pone.0026389.s002.docx]

**Supplementary Table S2.** Gene set enrichment analysis results

|  |  | **name** | **p-val** | **FDR** |
| --- | --- | --- | --- | --- |
| LA | MF1_3 | acetyltransferase activity | 1.68E-02 | 0.14 |
|  |  | amine transport | 0.00E+00 | 0.15 |
|  |  | amino sugar metabolic process | 0.00E+00 | 0.13 |
|  |  | cellular carbohydrate catabolic process | 1.44E-02 | 0.21 |
|  |  | cellular respiration | 1.44E-02 | 0.14 |
|  |  | coenzyme metabolic process | 3.66E-02 | 0.15 |
|  |  | fatty acid oxidation | 4.02E-03 | 0.13 |
|  |  | hydrogen ion transmembrane transporter activity | 2.48E-02 | 0.16 |
|  |  | inorganic cation transmembrane transporter activity | 1.86E-02 | 0.15 |
|  |  | kinase inhibitor activity | 0.00E+00 | 0.14 |
|  |  | kinase regulator activity | 6.26E-03 | 0.15 |
|  |  | mitochondrial envelope | 1.62E-02 | 0.15 |
|  |  | mitochondrial inner membrane | 3.98E-03 | 0.12 |
|  |  | mitochondrial lumen | 1.58E-02 | 0.13 |
|  |  | mitochondrial matrix | 1.58E-02 | 0.15 |
|  |  | mitochondrial membrane | 1.59E-02 | 0.15 |
|  |  | mitochondrial membrane part | 4.02E-03 | 0.12 |
|  |  | mitochondrial part | 3.98E-03 | 0.13 |
|  |  | mitochondrial respiratory chain | 0.00E+00 | 0.15 |
|  |  | mitochondrial ribosome | 0.00E+00 | 0.16 |
|  |  | mitochondrion | 3.05E-02 | 0.15 |
|  |  | n acetyltransferase activity | 4.05E-02 | 0.25 |
|  |  | organelle inner membrane | 1.21E-02 | 0.14 |
|  |  | protein folding | 2.08E-02 | 0.13 |
|  |  | protein kinase inhibitor activity | 0.00E+00 | 0.14 |
|  |  | protein kinase regulator activity | 0.00E+00 | 0.12 |
|  |  | protein serine threonine phosphatase activity | 3.70E-02 | 0.21 |
|  |  | regulation of g protein coupled receptor protein signaling pathway | 0.00E+00 | 0.12 |
|  |  | regulation of neurotransmitter levels | 4.05E-03 | 0.15 |
|  |  | response to hormone stimulus | 0.00E+00 | 0.12 |
|  |  | ribosomal subunit | 0.00E+00 | 0.12 |
|  |  | ribosome | 6.11E-03 | 0.14 |
|  |  | transferase activity transferring pentosyl groups | 4.48E-02 | 0.16 |
|  | MF1_12 | cofactor biosynthetic process | 0.00E+00 | 0.13 |
|  |  | contractile fiber | 0.00E+00 | 0.16 |
|  |  | contractile fiber part | 0.00E+00 | 0.13 |
|  |  | mitochondrial lumen | 0.00E+00 | 0.24 |
|  |  | mitochondrial ribosome | 0.00E+00 | 0.18 |
|  |  | organellar ribosome | 0.00E+00 | 0.22 |
|  |  | response to other organism | 0.00E+00 | 0.16 |
|  |  | response to virus | 0.00E+00 | 0.15 |
|  |  | ribosomal subunit | 0.00E+00 | 0.18 |
|  | SA_12 | acid amino acid ligase activity | 0.00E+00 | 0.22 |
|  |  | ligase activity | 0.00E+00 | 0.25 |
|  |  | mitochondrion | 0.00E+00 | 0.25 |
|  |  | protein kinase regulator activity | 0.00E+00 | 0.24 |
|  |  | small protein conjugating enzyme activity | 0.00E+00 | 0.24 |
| RA | MF1_3 | anion cation symporter activity | 1.91E-02 | 0.14 |
|  |  | anion transport | 1.64E-02 | 0.12 |
|  |  | calcium mediated signaling | 8.03E-03 | 0.08 |
|  |  | carbohydrate binding | 0.00E+00 | 0.09 |
|  |  | carbohydrate transmembrane transporter activity | 3.98E-03 | 0.09 |
|  |  | carbohydrate transport | 0.00E+00 | 0.01 |
|  |  | carboxylesterase activity | 0.00E+00 | 0.04 |
|  |  | cell activation | 0.00E+00 | 0.18 |
|  |  | cell matrix adhesion | 0.00E+00 | 0.05 |
|  |  | cell substrate adhesion | 0.00E+00 | 0.01 |
|  |  | cellular defense response | 2.61E-02 | 0.23 |
|  |  | defense response | 0.00E+00 | 0.17 |
|  |  | enzyme linked receptor protein signaling pathway | 0.00E+00 | 0.14 |
|  |  | establishment and or maintenance of cell polarity | 1.26E-02 | 0.09 |
|  |  | exonuclease activity | 0.00E+00 | 0.09 |
|  |  | extracellular region | 0.00E+00 | 0.16 |
|  |  | extracellular region part | 0.00E+00 | 0.17 |
|  |  | extracellular space | 0.00E+00 | 0.08 |
|  |  | glycerophospholipid biosynthetic process | 0.00E+00 | 0.12 |
|  |  | growth factor binding | 0.00E+00 | 0.04 |
|  |  | hormone metabolic process | 0.00E+00 | 0.05 |
|  |  | hormone receptor binding | 2.79E-02 | 0.20 |
|  |  | hydrolase activity acting on ester bonds | 0.00E+00 | 0.12 |
|  |  | inflammatory response | 0.00E+00 | 0.17 |
|  |  | inorganic anion transport | 0.00E+00 | 0.02 |
|  |  | insulin receptor signaling pathway | 0.00E+00 | 0.04 |
|  |  | integrin binding | 3.37E-02 | 0.19 |
|  |  | lipid biosynthetic process | 0.00E+00 | 0.19 |
|  |  | mesoderm development | 0.00E+00 | 0.01 |
|  |  | muscle cell differentiation | 1.82E-02 | 0.14 |
|  |  | myoblast differentiation | 2.26E-02 | 0.10 |
|  |  | negative regulation of binding | 0.00E+00 | 0.01 |
|  |  | negative regulation of cellular protein metabolic process | 1.78E-02 | 0.17 |
|  |  | negative regulation of dna binding | 0.00E+00 | 0.01 |
|  |  | negative regulation of growth | 1.01E-02 | 0.12 |
|  |  | negative regulation of metabolic process | 0.00E+00 | 0.21 |
|  |  | negative regulation of multicellular organismal process | 6.20E-03 | 0.10 |
|  |  | negative regulation of protein metabolic process | 1.82E-02 | 0.19 |
|  |  | negative regulation of signal transduction | 3.53E-02 | 0.20 |
|  |  | nuclear hormone receptor binding | 0.00E+00 | 0.17 |
|  |  | phospholipid biosynthetic process | 0.00E+00 | 0.08 |
|  |  | phosphoric diester hydrolase activity | 6.07E-03 | 0.14 |
|  |  | phosphoric ester hydrolase activity | 1.02E-02 | 0.23 |
|  |  | protease inhibitor activity | 1.02E-02 | 0.12 |
|  |  | protein complex binding | 0.00E+00 | 0.08 |
|  |  | protein tyrosine kinase activity | 0.00E+00 | 0.01 |
|  |  | protein tyrosine phosphatase activity | 0.00E+00 | 0.06 |
|  |  | regulation of binding | 0.00E+00 | 0.11 |
|  |  | regulation of cell migration | 3.99E-03 | 0.06 |
|  |  | regulation of dna binding | 0.00E+00 | 0.07 |
|  |  | regulation of multicellular organismal process | 0.00E+00 | 0.16 |
|  |  | regulation of response to stimulus | 0.00E+00 | 0.08 |
|  |  | regulation of transcription factor activity | 0.00E+00 | 0.09 |
|  |  | response to external stimulus | 0.00E+00 | 0.12 |
|  |  | response to stress | 0.00E+00 | 0.23 |
|  |  | response to wounding | 0.00E+00 | 0.04 |
|  |  | secretory granule | 3.33E-02 | 0.18 |
|  |  | skeletal development | 0.00E+00 | 0.07 |
|  |  | skeletal muscle development | 2.73E-02 | 0.15 |
|  |  | soluble fraction | 0.00E+00 | 0.02 |
|  |  | steroid biosynthetic process | 3.65E-02 | 0.17 |
|  |  | striated muscle development | 3.29E-02 | 0.17 |
|  |  | structural constituent of cytoskeleton | 0.00E+00 | 0.02 |
|  |  | sugar binding | 0.00E+00 | 0.04 |
|  |  | tissue development | 0.00E+00 | 0.18 |
|  |  | transcription repressor activity | 0.00E+00 | 0.22 |
|  |  | transforming growth factor beta receptor signaling pathway | 6.17E-03 | 0.11 |
|  |  | transmembrane receptor activity | 0.00E+00 | 0.15 |
|  |  | transmembrane receptor protein kinase activity | 0.00E+00 | 0.01 |
|  |  | transmembrane receptor protein phosphatase activity | 1.01E-02 | 0.15 |
|  |  | transmembrane receptor protein serine threonine kinase signaling | 3.88E-02 | 0.19 |
|  |  | transmembrane receptor protein tyrosine kinase activity | 0.00E+00 | 0.04 |
|  |  | transmembrane receptor protein tyrosine kinase signaling pathway | 8.02E-03 | 0.18 |
|  |  | wound healing | 6.01E-03 | 0.21 |
|  | MF1_12 | carbohydrate kinase activity | 0.00E+00 | 0.20 |
|  |  | chloride channel activity | 2.81E-02 | 0.19 |
|  |  | exonuclease activity | 2.72E-02 | 0.13 |
|  |  | hormone metabolic process | 0.00E+00 | 0.24 |
|  |  | regulation of defense response | 0.00E+00 | 0.13 |
|  |  | steroid biosynthetic process | 0.00E+00 | 0.17 |
|  |  | transforming growth factor beta receptor signaling pathway | 0.00E+00 | 0.21 |
|  | SA_12 | anion transport | 0.00E+00 | 0.24 |
|  |  | inorganic anion transport | 0.00E+00 | 0.04 |
