# Supplementary material for: Systematic Analysis of Gene Expression Differences between Left and Right Atria in Different Mouse Strains and in Human Atrial Tissue
Source: PLoS One. 2011 Oct 19;6(10):e26389. doi: 10.1371/journal.pone.0026389 (PMC3198471; doi:10.1371/journal.pone.0026389)
Supplement: Table S3 — FaTiGo results (Biological Process; Molecular Function, Cellular Compartment). (DOCX) [file pone.0026389.s003.docx]

**Supplementary Table S3**. FaTiGo results (Biological Process; Molecular Function, Cellular Compartment)

|  |  |  | **p value** | |
| --- | --- | --- | --- | --- |
| **Cat** | **Term** | **Definition** | **LA** | **RA** |
| BP | GO:0006936 | muscle contraction | 1.93E-04 | n.s. |
| BP | GO:0006937 | regulation of muscle contraction | 1.20E-03 | n.s. |
| BP | GO:0010811 | positive regulation of cell-substrate adhesion | 5.03E-03 | n.s. |
| BP | GO:0001568 | blood vessel development | 6.61E-04 | 1.16E-05 |
| BP | GO:0001944 | vasculature development | 6.61E-04 | 3.21E-06 |
| BP | GO:0007155 | cell adhesion | 1.85E-03 | 1.33E-04 |
| BP | GO:0009605 | response to external stimulus | 1.85E-03 | 1.67E-05 |
| BP | GO:0048514 | blood vessel morphogenesis | 1.85E-03 | 5.55E-05 |
| BP | GO:0031589 | cell-substrate adhesion | 7.08E-03 | 1.76E-03 |
| BP | GO:0048513 | organ development | n.s. | 8.43E-15 |
| BP | GO:0030154 | cell differentiation | n.s. | 1.91E-12 |
| BP | GO:0050793 | regulation of developmental process | n.s. | 2.43E-10 |
| BP | GO:0010033 | response to organic substance | n.s. | 1.31E-09 |
| BP | GO:0009653 | anatomical structure morphogenesis | n.s. | 3.30E-09 |
| BP | GO:0042221 | response to chemical stimulus | n.s. | 3.73E-09 |
| BP | GO:0040008 | regulation of growth | n.s. | 2.79E-08 |
| BP | GO:0008283 | cell proliferation | n.s. | 5.52E-08 |
| BP | GO:0048523 | negative regulation of cellular process | n.s. | 5.52E-08 |
| BP | GO:0007507 | heart development | n.s. | 3.21E-07 |
| BP | GO:0009887 | organ morphogenesis | n.s. | 6.17E-07 |
| BP | GO:0001558 | regulation of cell growth | n.s. | 7.05E-07 |
| BP | GO:0048468 | cell development | n.s. | 7.78E-07 |
| BP | GO:0009611 | response to wounding | n.s. | 1.55E-06 |
| BP | GO:0006950 | response to stress | n.s. | 3.17E-06 |
| BP | GO:0009888 | tissue development | n.s. | 3.21E-06 |
| BP | GO:0016049 | cell growth | n.s. | 4.14E-06 |
| BP | GO:0042127 | regulation of cell proliferation | n.s. | 5.10E-06 |
| BP | GO:0008219 | cell death | n.s. | 1.07E-05 |
| BP | GO:0008361 | regulation of cell size | n.s. | 1.21E-05 |
| BP | GO:0007399 | nervous system development | n.s. | 1.39E-05 |
| BP | GO:0008015 | blood circulation | n.s. | 2.45E-05 |
| BP | GO:0042692 | muscle cell differentiation | n.s. | 3.37E-05 |
| BP | GO:0007167 | enzyme linked receptor protein signaling pathway | n.s. | 4.00E-05 |
| BP | GO:0007517 | muscle organ development | n.s. | 6.22E-05 |
| BP | GO:0045595 | regulation of cell differentiation | n.s. | 7.74E-05 |
| BP | GO:0006915 | apoptosis | n.s. | 8.40E-05 |
| BP | GO:0009725 | response to hormone stimulus | n.s. | 8.43E-05 |
| BP | GO:0012501 | programmed cell death | n.s. | 8.62E-05 |
| BP | GO:0008285 | negative regulation of cell proliferation | n.s. | 1.08E-04 |
| BP | GO:0019752 | carboxylic acid metabolic process | n.s. | 1.19E-04 |
| BP | GO:0006082 | organic acid metabolic process | n.s. | 1.22E-04 |
| BP | GO:0001503 | ossification | n.s. | 1.30E-04 |
| BP | GO:0048545 | response to steroid hormone stimulus | n.s. | 1.38E-04 |
| BP | GO:0002526 | acute inflammatory response | n.s. | 1.40E-04 |
| BP | GO:0048732 | gland development | n.s. | 1.56E-04 |
| BP | GO:0060348 | bone development | n.s. | 1.94E-04 |
| BP | GO:0001501 | skeletal system development | n.s. | 2.46E-04 |
| BP | GO:0007568 | aging | n.s. | 2.46E-04 |
| BP | GO:0042592 | homeostatic process | n.s. | 2.46E-04 |
| BP | GO:0016055 | Wnt receptor signaling pathway | n.s. | 3.14E-04 |
| BP | GO:0042981 | regulation of apoptosis | n.s. | 3.14E-04 |
| BP | GO:0006547 | histidine metabolic process | n.s. | 3.24E-04 |
| BP | GO:0006955 | immune response | n.s. | 3.24E-04 |
| BP | GO:0043067 | regulation of programmed cell death | n.s. | 3.33E-04 |
| BP | GO:0001101 | response to acid | n.s. | 3.46E-04 |
| BP | GO:0010243 | response to organic nitrogen | n.s. | 4.02E-04 |
| BP | GO:0035295 | tube development | n.s. | 4.02E-04 |
| BP | GO:0010646 | regulation of cell communication | n.s. | 4.67E-04 |
| BP | GO:0043200 | response to amino acid stimulus | n.s. | 4.67E-04 |
| BP | GO:0045597 | positive regulation of cell differentiation | n.s. | 4.67E-04 |
| BP | GO:0050880 | regulation of blood vessel size | n.s. | 4.67E-04 |
| BP | GO:0048878 | chemical homeostasis | n.s. | 4.88E-04 |
| BP | GO:0009892 | negative regulation of metabolic process | n.s. | 4.88E-04 |
| BP | GO:0035265 | organ growth | n.s. | 4.88E-04 |
| BP | GO:0045787 | positive regulation of cell cycle | n.s. | 4.88E-04 |
| BP | GO:0045833 | negative regulation of lipid metabolic process | n.s. | 4.93E-04 |
| BP | GO:0006954 | inflammatory response | n.s. | 5.09E-04 |
| BP | GO:0007169 | transmembrane receptor protein tyrosine kinase signaling | n.s. | 5.51E-04 |
| BP | GO:0006953 | acute-phase response | n.s. | 5.58E-04 |
| BP | GO:0014075 | response to amine stimulus | n.s. | 5.58E-04 |
| BP | GO:0042310 | vasoconstriction | n.s. | 5.58E-04 |
| BP | GO:0045471 | response to ethanol | n.s. | 5.58E-04 |
| BP | GO:0002684 | positive regulation of immune system process | n.s. | 6.06E-04 |
| BP | GO:0043066 | negative regulation of apoptosis | n.s. | 6.06E-04 |
| BP | GO:0043069 | negative regulation of programmed cell death | n.s. | 6.38E-04 |
| BP | GO:0001649 | osteoblast differentiation | n.s. | 7.21E-04 |
| BP | GO:0009620 | response to fungus | n.s. | 7.21E-04 |
| BP | GO:0048562 | embryonic organ morphogenesis | n.s. | 7.21E-04 |
| BP | GO:0060548 | negative regulation of cell death | n.s. | 7.21E-04 |
| BP | GO:0001525 | angiogenesis | n.s. | 8.28E-04 |
| BP | GO:0051384 | response to glucocorticoid stimulus | n.s. | 8.28E-04 |
| BP | GO:0009880 | embryonic pattern specification | n.s. | 9.08E-04 |
| BP | GO:0046620 | regulation of organ growth | n.s. | 9.08E-04 |
| BP | GO:0003007 | heart morphogenesis | n.s. | 9.18E-04 |
| BP | GO:0016202 | regulation of striated muscle tissue development | n.s. | 9.18E-04 |
| BP | GO:0002682 | regulation of immune system process | n.s. | 9.40E-04 |
| BP | GO:0031960 | response to corticosteroid stimulus | n.s. | 1.05E-03 |
| BP | GO:0009790 | embryo development | n.s. | 1.14E-03 |
| BP | GO:0051789 | response to protein stimulus | n.s. | 1.19E-03 |
| BP | GO:0030879 | mammary gland development | n.s. | 1.28E-03 |
| BP | GO:0002253 | activation of immune response | n.s. | 1.33E-03 |
| BP | GO:0006633 | fatty acid biosynthetic process | n.s. | 1.33E-03 |
| BP | GO:0007389 | pattern specification process | n.s. | 1.33E-03 |
| BP | GO:0006873 | cellular ion homeostasis | n.s. | 1.38E-03 |
| BP | GO:0007267 | cell-cell signaling | n.s. | 1.38E-03 |
| BP | GO:0035282 | segmentation | n.s. | 1.38E-03 |
| BP | GO:0050801 | ion homeostasis | n.s. | 1.38E-03 |
| BP | GO:0001947 | heart looping | n.s. | 1.44E-03 |
| BP | GO:0007519 | skeletal muscle tissue development | n.s. | 1.44E-03 |
| BP | GO:0009063 | cellular amino acid catabolic process | n.s. | 1.61E-03 |
| BP | GO:0042311 | vasodilation | n.s. | 1.61E-03 |
| BP | GO:0048568 | embryonic organ development | n.s. | 1.61E-03 |
| BP | GO:0048589 | developmental growth | n.s. | 1.61E-03 |
| BP | GO:0055082 | cellular chemical homeostasis | n.s. | 1.61E-03 |
| BP | GO:0009968 | negative regulation of signal transduction | n.s. | 1.68E-03 |
| BP | GO:0006952 | defense response | n.s. | 1.82E-03 |
| BP | GO:0009966 | regulation of signal transduction | n.s. | 1.82E-03 |
| BP | GO:0048747 | muscle fiber development | n.s. | 1.82E-03 |
| BP | GO:0014829 | vascular smooth muscle contraction | n.s. | 1.86E-03 |
| BP | GO:0032808 | lacrimal gland development | n.s. | 1.86E-03 |
| BP | GO:0002520 | immune system development | n.s. | 1.87E-03 |
| BP | GO:0007610 | behavior | n.s. | 1.96E-03 |
| BP | GO:0006468 | protein amino acid phosphorylation | n.s. | 2.02E-03 |
| BP | GO:0000904 | cell morphogenesis involved in differentiation | n.s. | 2.10E-03 |
| BP | GO:0035108 | limb morphogenesis | n.s. | 2.10E-03 |
| BP | GO:0006816 | calcium ion transport | n.s. | 2.19E-03 |
| BP | GO:0060173 | limb development | n.s. | 2.34E-03 |
| BP | GO:0048699 | generation of neurons | n.s. | 2.36E-03 |
| BP | GO:0019886 | antigen processing and presentation via MHc class II | n.s. | 2.42E-03 |
| BP | GO:0035051 | cardiac cell differentiation | n.s. | 2.42E-03 |
| BP | GO:0048646 | anatomical structure formation involved in morphogenesis | n.s. | 2.42E-03 |
| BP | GO:0050778 | positive regulation of immune response | n.s. | 2.57E-03 |
| BP | GO:0030509 | BMp signaling pathway | n.s. | 2.65E-03 |
| BP | GO:0016477 | cell migration | n.s. | 2.96E-03 |
| BP | GO:0051605 | protein maturation by peptide bond cleavage | n.s. | 2.96E-03 |
| BP | GO:0001660 | fever | n.s. | 3.05E-03 |
| BP | GO:0002675 | positive regulation of acute inflammatory response | n.s. | 3.05E-03 |
| BP | GO:0043616 | keratinocyte proliferation | n.s. | 3.05E-03 |
| BP | GO:0048259 | regulation of receptor-mediated endocytosis | n.s. | 3.05E-03 |
| BP | GO:0048557 | embryonic digestive tract morphogenesis | n.s. | 3.05E-03 |
| BP | GO:0050995 | negative regulation of lipid catabolic process | n.s. | 3.05E-03 |
| BP | GO:0000902 | cell morphogenesis | n.s. | 3.12E-03 |
| BP | GO:0048754 | branching morphogenesis of a tube | n.s. | 3.22E-03 |
| BP | GO:0002541 | activation of plasma proteins involved in acute inflammatory response | n.s. | 3.58E-03 |
| BP | GO:0055072 | iron ion homeostasis | n.s. | 3.58E-03 |
| BP | GO:0015674 | di-,tri-valent inorganic cation transport | n.s. | 3.60E-03 |
| BP | GO:0006898 | receptor-mediated endocytosis | n.s. | 3.89E-03 |
| BP | GO:0043154 | negative regulation of caspase activity | n.s. | 3.89E-03 |
| BP | GO:0001763 | morphogenesis of a branching structure | n.s. | 4.07E-03 |
| BP | GO:0051346 | negative regulation of hydrolase activity | n.s. | 4.16E-03 |
| BP | GO:0016064 | immunoglobulin mediated immune response | n.s. | 4.59E-03 |
| BP | GO:0000578 | embryonic axis specification | n.s. | 4.65E-03 |
| BP | GO:0001502 | cartilage condensation | n.s. | 4.65E-03 |
| BP | GO:0001756 | somitogenesis | n.s. | 4.65E-03 |
| BP | GO:0002504 | antigen processing and presentation via MHc class II | n.s. | 4.65E-03 |
| BP | GO:0006811 | ion transport | n.s. | 4.65E-03 |
| BP | GO:0010466 | negative regulation of peptidase activity | n.s. | 4.65E-03 |
| BP | GO:0031032 | actomyosin structure organization | n.s. | 4.65E-03 |
| BP | GO:0031649 | heat generation | n.s. | 4.65E-03 |
| BP | GO:0033574 | response to testosterone stimulus | n.s. | 4.65E-03 |
| BP | GO:0050776 | regulation of immune response | n.s. | 4.65E-03 |
| BP | GO:0022008 | neurogenesis | n.s. | 4.66E-03 |
| BP | GO:0030182 | neuron differentiation | n.s. | 4.67E-03 |
| BP | GO:0019724 | b cell mediated immunity | n.s. | 4.73E-03 |
| BP | GO:0030001 | metal ion transport | n.s. | 4.73E-03 |
| BP | GO:0030326 | embryonic limb morphogenesis | n.s. | 4.73E-03 |
| BP | GO:0006812 | cation transport | n.s. | 5.14E-03 |
| BP | GO:0006641 | triglyceride metabolic process | n.s. | 5.69E-03 |
| BP | GO:0006631 | fatty acid metabolic process | n.s. | 5.84E-03 |
| BP | GO:0014031 | mesenchymal cell development | n.s. | 6.05E-03 |
| BP | GO:0030324 | lung development | n.s. | 6.38E-03 |
| BP | GO:0006805 | xenobiotic metabolic process | n.s. | 6.40E-03 |
| BP | GO:0007157 | heterophilic cell-cell adhesion | n.s. | 6.40E-03 |
| BP | GO:0008306 | associative learning | n.s. | 6.40E-03 |
| BP | GO:0019725 | cellular homeostasis | n.s. | 6.40E-03 |
| BP | GO:0034097 | response to cytokine stimulus | n.s. | 6.40E-03 |
| BP | GO:0034381 | lipoprotein particle clearance | n.s. | 6.40E-03 |
| BP | GO:0045931 | positive regulation of mitotic cell cycle | n.s. | 6.40E-03 |
| BP | GO:0030323 | respiratory tube development | n.s. | 6.63E-03 |
| BP | GO:0031100 | organ regeneration | n.s. | 6.63E-03 |
| BP | GO:0048762 | mesenchymal cell differentiation | n.s. | 6.63E-03 |
| BP | GO:0045596 | negative regulation of cell differentiation | n.s. | 6.68E-03 |
| BP | GO:0006629 | lipid metabolic process | n.s. | 6.75E-03 |
| BP | GO:0043434 | response to peptide hormone stimulus | n.s. | 6.85E-03 |
| BP | GO:0045926 | negative regulation of growth | n.s. | 6.85E-03 |
| BP | GO:0046395 | carboxylic acid catabolic process | n.s. | 6.85E-03 |
| BP | GO:0035239 | tube morphogenesis | n.s. | 6.97E-03 |
| BP | GO:0060541 | respiratory system development | n.s. | 6.97E-03 |
| BP | GO:0051128 | regulation of cellular component organization | n.s. | 7.67E-03 |
| BP | GO:0030111 | regulation of Wnt receptor signaling pathway | n.s. | 7.82E-03 |
| BP | GO:0007611 | learning or memory | n.s. | 8.29E-03 |
| BP | GO:0006959 | humoral immune response | n.s. | 8.31E-03 |
| BP | GO:0051246 | regulation of protein metabolic process | n.s. | 8.31E-03 |
| BP | GO:0009410 | response to xenobiotic stimulus | n.s. | 8.37E-03 |
| BP | GO:0030279 | negative regulation of ossification | n.s. | 8.37E-03 |
| BP | GO:0033700 | phospholipid efflux | n.s. | 8.37E-03 |
| BP | GO:0044255 | cellular lipid metabolic process | n.s. | 8.37E-03 |
| BP | GO:0046653 | tetrahydrofolate metabolic process | n.s. | 8.37E-03 |
| BP | GO:0048535 | lymph node development | n.s. | 8.37E-03 |
| BP | GO:0006916 | anti-apoptosis | n.s. | 8.56E-03 |
| BP | GO:0042326 | negative regulation of phosphorylation | n.s. | 8.99E-03 |
| BP | GO:0045807 | positive regulation of endocytosis | n.s. | 8.99E-03 |
| BP | GO:0050678 | regulation of epithelial cell proliferation | n.s. | 9.05E-03 |
| BP | GO:0042110 | t cell activation | n.s. | 9.10E-03 |
| BP | GO:0009890 | negative regulation of biosynthetic process | n.s. | 9.25E-03 |
| BP | GO:0001775 | cell activation | n.s. | 9.66E-03 |
| BP | GO:0001666 | response to hypoxia | n.s. | 9.71E-03 |
| BP | GO:0007010 | cytoskeleton organization | n.s. | 9.71E-03 |
| BP | GO:0016042 | lipid catabolic process | n.s. | 9.71E-03 |
| MF | GO:0008009 | chemokine activity | 4.08E-04 | n.s. |
| MF | GO:0001664 | G-protein-coupled receptor binding | 2.88E-03 | n.s. |
| MF | GO:0005126 | cytokine receptor binding | 5.05E-03 | n.s. |
| MF | GO:0005509 | calcium ion binding | 2.72E-06 | 9.99E-03 |
| MF | GO:0030246 | carbohydrate binding | 1.34E-03 | 1.50E-03 |
| MF | GO:0019838 | growth factor binding | 1.73E-03 | 2.42E-06 |
| MF | GO:0005102 | receptor binding | 3.83E-03 | 3.28E-04 |
| MF | GO:0005520 | insulin-like growth factor binding | 8.34E-03 | 5.65E-07 |
| MF | GO:0031406 | carboxylic acid binding | n.s. | 2.43E-05 |
| MF | GO:0019842 | vitamin binding | n.s. | 1.67E-04 |
| MF | GO:0030247 | polysaccharide binding | n.s. | 3.28E-04 |
| MF | GO:0031994 | insulin-like growth factor i binding | n.s. | 4.01E-04 |
| MF | GO:0005539 | glycosaminoglycan binding | n.s. | 4.92E-04 |
| MF | GO:0016597 | amino acid binding | n.s. | 9.78E-04 |
| MF | GO:0004869 | cysteine-type endopeptidase inhibitor activity | n.s. | 1.29E-03 |
| MF | GO:0008201 | heparin binding | n.s. | 2.77E-03 |
| MF | GO:0042803 | protein homodimerization activity | n.s. | 2.77E-03 |
| MF | GO:0008095 | inositol-1,4,5-trisphosphate receptor activity | n.s. | 5.86E-03 |
| MF | GO:0005072 | transforming growth factor beta receptor | n.s. | 9.99E-03 |
| MF | GO:0005545 | phosphatidylinositol binding | n.s. | 9.99E-03 |
| CC | GO:0030016 | myofibril | 3.21E-07 | n.s. |
| CC | GO:0044420 | extracellular matrix part | 2.06E-05 | n.s. |
| CC | GO:0005604 | basement membrane | 3.22E-04 | n.s. |
| CC | GO:0044421 | extracellular region part | 2.42E-15 | 5.64E-10 |
| CC | GO:0005615 | extracellular space | 1.33E-14 | 1.21E-10 |
| CC | GO:0031012 | extracellular matrix | 2.85E-11 | 1.19E-04 |
| CC | GO:0005578 | proteinaceous extracellular matrix | 2.54E-09 | 2.11E-04 |
| CC | GO:0043292 | contractile fiber | 4.74E-07 | 2.15E-03 |
| CC | GO:0016529 | sarcoplasmic reticulum | 8.30E-03 | 5.49E-03 |
| CC | GO:0016528 | sarcoplasm | 9.28E-03 | 6.28E-03 |
| CC | GO:0044459 | plasma membrane part | n.s. | 5.64E-10 |
| CC | GO:0009986 | cell surface | n.s. | 9.00E-08 |
| CC | GO:0005625 | soluble fraction | n.s. | 8.78E-05 |
| CC | GO:0000267 | cell fraction | n.s. | 9.95E-05 |
| CC | GO:0019897 | extrinsic to plasma membrane | n.s. | 9.95E-05 |
| CC | GO:0042995 | cell projection | n.s. | 1.02E-04 |
| CC | GO:0009897 | external side of plasma membrane | n.s. | 1.19E-04 |
| CC | GO:0019898 | extrinsic to membrane | n.s. | 1.19E-04 |
| CC | GO:0043005 | neuron projection | n.s. | 5.30E-04 |
| CC | GO:0005834 | heterotrimeric G-protein complex | n.s. | 7.74E-04 |
| CC | GO:0043204 | perikaryon | n.s. | 1.52E-03 |
| CC | GO:0016323 | basolateral plasma membrane | n.s. | 3.29E-03 |
| CC | GO:0043197 | dendritic spine | n.s. | 3.31E-03 |
| CC | GO:0005783 | endoplasmic reticulum | n.s. | 3.64E-03 |
| CC | GO:0005626 | insoluble fraction | n.s. | 5.44E-03 |
| CC | GO:0005791 | rough endoplasmic reticulum | n.s. | 5.44E-03 |
| CC | GO:0031225 | anchored to membrane | n.s. | 5.44E-03 |
| CC | GO:0015629 | actin cytoskeleton | n.s. | 6.28E-03 |
| CC | GO:0005614 | interstitial matrix | n.s. | 6.30E-03 |
| CC | GO:0030425 | dendrite | n.s. | 6.30E-03 |
| CC | GO:0034361 | very-low-density lipoprotein particle | n.s. | 6.30E-03 |
| CC | GO:0031410 | cytoplasmic vesicle | n.s. | 6.39E-03 |
| CC | GO:0016023 | cytoplasmic membrane-bounded vesicle | n.s. | 6.73E-03 |
| CC | GO:0005624 | membrane fraction | n.s. | 6.74E-03 |
| CC | GO:0042613 | MHc class Ii protein complex | n.s. | 7.75E-03 |
| CC | GO:0005768 | endosome | n.s. | 8.73E-03 |
| CC | GO:0031988 | membrane-bounded vesicle | n.s. | 8.73E-03 |
