# Supplementary material for: Systematic Analysis of Gene Expression Differences between Left and Right Atria in Different Mouse Strains and in Human Atrial Tissue
Source: PLoS One. 2011 Oct 19;6(10):e26389. doi: 10.1371/journal.pone.0026389 (PMC3198471; doi:10.1371/journal.pone.0026389)
Supplement: Table S4 — RT-qPCR primers for selected murine and orthologous human genes. (DOCX) [file pone.0026389.s004.docx]

**Supplementary Table S4.** RT-qPCR primers for selected murine and orthologous human genes.

| **Organism** | **Gene** | **Transcript** | **Forward (5‘-3‘)** | **Reverse (5‘-3‘)** |
| --- | --- | --- | --- | --- |
| Mouse | *Bmp10* | NM_009756 | GACTCCTGGATCATCGCTCCTC | CAAGGCCTGAATAATTGCGTGTT |
|  | *Adm* | NM_009627 | GACGTGAATGTCTCAGCAAGGTG | GGCGATAATCAGGCGCTCTC |
|  | *Ppp1r1b* | NM_144828 | ATGGACTGGCAGAGGCTGCT | GCTGGAGGACAAGGAGAACCAG |
|  | *Mapk10* | NM_009158 | TGAAAGGGAGCACACCATCG | CGAGGGCTGGCCTTTGACTA |
|  |  | NM_001081567.1 |  |  |
|  | *Kcnc4* | NM_145922 | GCTGTCCTCTTGTGTGTGTGTCTG | TTGGTACTCGAGCACAGCATGA |
|  | *Irx3* | NM_008393 | CAGATCGCTGTAGTGCCTTGGA | GCGTCCAGATGGTTCTGTGG |
|  | *Pitx2c* | NM_001042502 | GAGGTGCATACAATCTCCGATA | TGCCGCTTCTTCTTGGAC |
|  | *Scn4b* | NM_001013390 | CATGGTTTAGTCCTCTGGCTTGG | CACGGCCCACCACTGTATTG |
|  | *Ryr3* | NM_177652 | TGTGTTCCAGAAGGACAACTCCA | GCAAGTGCAGTGTCATGACGTAAG |
|  | *Gapdh* | NM_008084.2 | CCAATGTGTCCGTCGTGGAT | TGCCTGCTTCACCACCTTCT |
| Human | *Bmp10* | NM_014482.1 | AAGCCTATGAATGCCGTGGTG | AGGCCTGGATAATTGCATGCTT |
|  | *Adm* | NM_001124 | CGTGAATGTCTCAGCGAGGTG | CCTTCTTCCACACAGGAGGTAATCA |
|  | *Ppp1r1b* | NM_032192.2 | TTCCCTTAATCACCCTTGCTCCT | GCATTCCTGGCATCAAAGCA |
|  |  | NM_181505.2 |  |  |
|  | *Mapk10* | NM_002753.3 | TTGCACTCTGACCATGTTGGTG | TCATCAACCATCCACTTCCTGTCT |
|  |  | NM_138980.2 |  |  |
|  |  | NM_138981.2 |  |  |
|  |  | NM_138982.2 |  |  |
|  | *Kcnc4* | NM_001039574.2 | TGTCTGCATGCCTCCAACTGT | TGCAGGTGCTCACACAGCAG |
|  |  | NM_004978.4 |  |  |
|  | *Irx3* | NM_024336.2 | CCGCATTCGCTGTGAGGTT | ACACACACAAAGGCAGACACGTT |
|  | *Pitx2c* | NM_000325.5 | CACCATCTCCGACACCTCCA | GCCCACGTCCTCATTCTTCC |
|  | *Pitx2* | NM_153427.1 | GCTTGCGAGCAAGGGAGTGTA | CATTGCATCCACCAGAGAAACTATTC |
|  |  | NM_153426.1 |  |  |
|  |  | NM_000325.5 |  |  |
|  | *Scn4b* | NM_001142348.1 | CCTCTGGCTTGGATGACAACAA | AGAGGCACTCGCACACCTGA |
|  |  | NM_001142349.1 |  |  |
|  |  | NM_174934.3 |  |  |
|  | *Ryr3* | NM_001036.3 | ATTTCTTCCCAGCCGGTGACT | TCCAGAATTGTCGCGCTTCTT |
|  | *Actb* | NM_001101.3 | TCAAGATCATTGCTCCTCCTGAG | ACATCTGCTGGAAGGTGGACA |
